# Small nucleolar RNA SNORD13H suppresses tumor progression via FBL-dependent 2′-O-methylation in hepatocellular carcinoma

**DOI:** 10.3389/fgene.2025.1620552

**Published:** 2025-08-21

**Authors:** Minglu Zhang, Jianbo He, Rao Fu, Guojian Bao, Yijun Lu, Zechuan Zhang, Jiawu Yan, Jialu Ding, Fei Yang, Beicheng Sun

**Affiliations:** ^1^ Nanjing Drum Tower Hospital, Affiliated Hospital of Medical School, Nanjing University, Nanjing, China; ^2^ Department of Hepatobiliary Surgery, The First Affiliated Hospital of Anhui Medical University, Hefei, China; ^3^ MOE Innovation Center for Basic Research in Tumor Immunotherapy, Hefei, China; ^4^ Anhui Province Key Laboratory of Tumor Immune Microenvironment and Immunotherapy, Hefei, China

**Keywords:** 2′-O-methylation, snoRNA, HCC, rRNA, RAS

## Abstract

**Introduction:**

Small nucleolar RNA (snoRNA) mediates RNA modifications, including 2′-O-methylation (Nm) and pseudouridine (Ψ), which has been proven to impact tumor progression. However, the role of snoRNA in the epigenetics of tumors remains poorly understood due to the lack of sufficiently effective experimental methods to identify snoRNA targets. Here, we identified SNORD13H, a C/D box snoRNA, as being downregulated in hepatocellular carcinoma (HCC), and its low expression was associated with HCC development.

**Methods:**

To elucidate specific roles of SNORD13H in HCC, we used a comprehensive array of methodologies, including flow cytometry, xenograft mouse model, reverse transcription at low dNTP concentration followed by PCR (RTL-P) assay, and surface sensing of translation (SUnSET) assay.

**Results:**

In this study, we first demonstrated that reduced SNORD13H serves as a biomarker for HCC, facilitating cellular proliferation. SNORD13H mediates 2′-O-methylations of 18S rRNA and RAS mRNA, thereby enhancing translation efficiency and regulating RAS protein levels in HCC. The diminution of SNORD13H activates the RAS pathway, contributing to the progression of HCC.

**Discussion:**

Our study establishes SNORD13H as a dual-function regulator in HCC progression. Furthermore, our findings indicate that SNORD13H is detectable in plasma, highlighting its potential utility in tumor screening.

## 1 Introduction

Tumors are heterogeneous entities composed of multiple genetic clones and diverse epigenetic markers. Tumor progression is significantly influenced by epigenetic regulation (Liu et al., 2020). Epigenetic modifications play a pivotal role in the acquisition of hallmark traits during tumor initiation and malignant progression (Hanahan, 2022). A novel feature of cancer cells is non-mutational epigenetic reprogramming. Moreover, tumor heterogeneity, driven by genetic, epigenetic, and metabolic diversity, contributes to treatment resistance and poses a major challenge to the efficacy of targeted therapies (Rovira-Clavé et al., 2022). Consequently, elucidating epigenetically driven intratumoral heterogeneity and its impact on tumor evolution has become a key priority in cancer research.

Hepatocellular carcinoma (HCC) exhibits a complex and multistage progression driven by diverse genetic and epigenetic alternations (Wilson et al., 2017). As one of the most prevalent and lethal malignancies within the digestive system, the efficacy of HCC treatment has been extremely constrained by tumor heterogeneity (Rumgay et al., 2022; Villanueva, 2019). Despite advances in therapeutic strategies for HCC, the 5-year survival rate remains dismally low (Wang et al., 2018). Early detection is crucial for reducing HCC mortality rates as it enables the application of potentially curative interventions (Sun et al., 2020). Currently, HCC is primarily diagnosed using typical imaging and elevated serum alpha-fetoprotein (AFP) levels, which lack sensitivity and accuracy for HCC screening, with approximately 30% of early-stage patients having normal AFP levels (Galle et al., 2019). Therefore, it is crucial to explore a new molecular biomarker for early HCC detection. Research on epigenetic processes in HCC is growing, showing its role in tumorigenesis and diagnosis (Yang et al., 2022).

With advancements in high-throughput sequencing, it is known that over 98% of the genome comprises noncoding genes (Lu et al., 2019). Noncoding RNA (ncRNA), a key part of epigenetics, has had its features and functions increasingly revealed. As an ancient and abundant family of ncRNA, small nucleolar RNA (snoRNA), ranging from 60 to 300 nucleotides in length, is classified into H/ACA box and C/D box snoRNA based on conserved sequences (Williams and Farzaneh, 2012). They perform pseudouridylation (Ψ) and 2′-O-methylation (Nm), respectively, in the nucleolus. As a reversible RNA post-transcriptional modification, 2′-O-methylation affects RNA processing, translation efficiency, and ribosomal biogenesis primarily targeting rRNA and mRNA. Its relevance to numerous human diseases, particularly cancer, is significant (Bratkovic et al., 2020). Growing evidence reveals that dysregulated 2′-O-methylation contributes to tumor progression through multiple mechanisms. 2′-O-Methylations on rRNA regulate its splicing and maturation (Cao et al., 2018), and inhibit ribosome translation (Yi et al., 2021). Moreover, 2′-O-methylation of mRNA strengthens its stability and suppresses ribosomal decoding, with reduced methylation often increasing protein synthesis (Elliott et al., 2019). This study explores how SNORD13H mediates 2′-O-methylation of RNA, revealing a novel methylation-dependent tumor development switch. As investigated previously, changes in snoRNA expression affect cell viability (Gong et al., 2017) and RNA modification, and regulate key signaling pathways such as MAPK, p53, and mTOR (Siprashvili et al., 2016; Su et al., 2021; Kan et al., 2021), which link to prognosis in cancer (Xu et al., 2014; Okugawa et al., 2017). Moreover, many snoRNAs have been extensively studied as potential tumor biomarkers, with their detectability and expression differences in serum or plasma (Wang K. et al., 2022). This systematic investigation of SNORD13H has revealed its multifaceted role in HCC pathogenesis through the regulation of 2′-O-methylation in both rRNA and oncogenic mRNA, and modulation of MAPK/ERK signaling activity. Findings demonstrate its clinical potential as a diagnostic biomarker for HCC screening. Significant inhibition of malignant phenotypes upon SNORD13H restoration underscores its therapeutic promise for HCC treatment strategies.

## 2 Materials and methods

### 2.1 Human samples

Human samples used in this work were totally supported by patients treated in the Drum Tower Hospital (Nanjing, China), which contained human tissues and blood samples. Informed consent was obtained from all patients included in this study. This research was approved by the Institutional Ethics Committee of Nanjing Drum Tower Hospital (2021-608-01). All samples were fresh-frozen and stored at −80 °C.

### 2.2 Cell culture and transfection

Human HCC cell lines (L-02, Hep3B, HepG2, MHCC-97L, MHCC-97H, MHCC-LM3, SMMC-7721, and Huh7) and 293T cells were purchased from the China Academy of Science Cell Bank. Cells for experiments were cultured in DMEM supplemented with 10% fetal bovine serum (FBS, Wisent, Saint Bruno, Canada) and routinely monitored for *mycoplasma*. SNORD13H was knocked out using the lentiviral-mediated CRISPR–Cas9 system. The sgRNA target sequences and protocol were obtained from the Zhang Lab website (http://www.genome-engineering.org). Lentivirus containing short hairpin RNA (shRNA) plasmids was used to knock down RAS and fibrillarin (FBL), whose targeting sequences were obtained from Sigma-Aldrich (Merck, Darmstadt, Germany). Detailed sequences are shown in Supplementary Table 1. Lentiviral packaging plasmids were selected in reference to a standard method. Lipo3000 and P3000 were purchased from Invitrogen (CA, United States), and polybrene was purchased from Sigma-Aldrich (Merck, Darmstadt, Germany). Viral packaging and infection were performed in accordance with the manufacturer’s instruction and usual protocol.

### 2.3 Cell proliferation, cell cycle, and apoptosis assay

Cell proliferation was monitored via the CCK-8 assay (Dojindo, Kumamoto, Japan) according to the product instructions. Clonogenic potential of HCC cells was determined through a colony formation assay. For cell cycle experiments, a PI Cell Cycle Kit (Vazyme, Nanjing, China) was used to stain DNA. In addition, Annexin V-FITC/PI from the Apoptosis Detection Kit (Vazyme, Nanjing, China) was used in the cell apoptosis assay. Flow cytometry was applied to analyze both the cell cycle and apoptotic cells. The specific operation was carried out according to the manufacturer’s instruction.

### 2.4 Animal model

The subcutaneous tumor-bearing mouse model was established using BALB/c nude mice, which were purchased from GemPharmatech Co. Ltd (Nanjing, China). Mice used in this research were kept in SPF environment at all times. There were six BALB/c nude mice (5–6 weeks old) in each group. HCC cells (2–3 × 10^5^) were injected subcutaneously into the inguinal area. Solid tumors at the inoculation sites formed approximately 10 days after injection, and tumor sizes were measured every 5 days until sacrifice. Tumor size and weight were measured and recorded for further analysis. All animal care and animal experiments were performed in accordance with the guidelines of ethical regulations and the Helsinki Declaration, and all treatments adhered to the rules of the Animal Care and Use Committee of the First Affiliated Hospital of Anhui Medical University (2024021904).

### 2.5 RNA extraction and quantitative real-time PCR (Q-PCR)

Total RNA was extracted using the TRIzol RNA extraction reagent (Invitrogen, CA, United States) following the manufacturer’s protocol. After measuring RNA concentration using the NanoDrop technology, equal amounts of RNA were reverse-transcribed to cDNA using HiScript II Q RT SuperMix for qPCR (Vazyme, Nanjing, China). The cDNA was then used for next quantitative real-time PCR with SYBR Green (Vazyme, Nanjing, China). Total plasma miRNA was isolated using the miRNeasy Serum/Plasma Kit (QIAGEN, Hilden, Germany). Reverse transcription and real-time quantitation were carried out with specific kits, such as the miRcute miRNA First-Strand cDNA Synthesis Kit (QIAGEN, Hilden, Germany) and the miRcute miRNA SYBR Green qPCR Detection Kit (QIAGEN, Hilden, Germany). RNA levels were calculated using U6 or β-actin as control. Primer sequences are detailed in Supplementary Table 2.

### 2.6 RTL-P assay for RNA 2′-O-methylation

Based on the previous publications, 2′-O-methylation levels were detected using reverse transcription at low dNTP concentrations followed by PCR (RTL-P) assay to quantify SNORD13H modification efficiency (Dong et al., 2012; Wu et al., 2020). The presence of 2′-O-methyl modifications impedes reverse transcription at low dNTP concentrations, creating a detectable difference compared to high dNTP conditions (Figure 3A). Based on established rRNA modification maps, 18S and 28S rRNAs were divided into five segments, each containing at least one known modification site, and corresponding primer sets (paired Fu, Fd, and R) were designed. The Fu primer amplifies across potential modification sites, whereas the Fd product serves as an internal control. High dNTP reactions provide additional controls for each sample. As reported in previous studies, reverse transcription was performed with 100 ng of RNA in a 25-μL reaction system. RTase M-MLV (RNase H-) (Takara, Kusatsu, Japan) and RNase Inhibitor (Takara, Kusatsu, Japan) were also added. In the following PCR step, Ex taq DNA polymerase (Takara, Kusatsu, Japan) and dNTP (Takara, Kusatsu, Japan) were mixed in a 10-μL reaction system with primers and products from the last step. The specific PCR process and details of experimental design were provided by Dong et al. (2012) and Wu et al. (2020). The products were subjected to agarose gel electrophoresis with 2% agarose gels and visualized through UV-trans-illumination. Primer sequences are shown in Supplementary Table 3.

### 2.7 Surface sensing of translation (SUnSET) assay

To display ribosomal translation efficiency more directly, the surface sensing of translation (SUnSET) technique was performed according to Yi et al. (2021) and Schmidt et al. (2009). As described previously, the experimental principle is labeling newly synthesized proteins with puromycin. Cells were treated with 335 μM CHX (Aladdin, Shanghai, China) for 15 min. Then, 91 μM puromycin was added to incubate cells at 37 °C for 5 min. Results were assessed using the Western blot assay with the anti-puromycin antibody (Sigma-Aldrich, Darmstadt, Germany).

### 2.8 Ribosomal RNA processing analysis

The procession of rRNA was simply measured using the qRT-PCR assay according to Cao et al. (2018). After extracting the total RNAs from HCC cells and reverse transcription, rRNA procession was verified using quantitative real-time PCR as usual. Gene-specific primers were designed (Supplementary Figure 4C), whose sequences are shown in Supplementary Table 4. The Ct values for primers 2/1 and b/a represent mature 18S and 28S rRNAs, respectively. Meanwhile, the primers targeting 5′ ETS-18S (primers 4/3 and 6/5) and ITS2-28S (primers d/c and f/e) represent unprocessed rRNA. The ratios of the Ct value (unprocessed group) to the Ct value (mature group) were averaged, meaning for the fraction of unprocessed rRNA. Primer pairs 4/3 over 2/1 and primer pairs 6/5 over 2/1 were for 18S rRNA, and primer pairs d/c over b/a and primer pairs f/e over b/a were for 28S rRNA.

### 2.9 Immunoblotting and antibodies

Immunoblotting was carried out with standard methods and protocol using commercially available antibodies. Antibodies used in this study are listed in Supplementary Table 5.

### 2.10 Immunohistochemistry and HE staining

Tumor samples from the subcutaneous tumor-bearing mouse model were fixed in 4% formaldehyde and sent to Servicebio (Wuhan, China) for paraffin-embedded sections. The hematoxylin and eosin (HE) staining experiment was performed by Servicebio (Wuhan, China). Immunohistochemistry was performed using generic methods and standard protocol with a commercial immunohistochemical staining kit (Maixin Biotech, Ltd., Fuzhou, China). Antibodies are commercially available, including anti-Ki-67 antibody (Abcam, Cambridge, United Kingdom) and anti-RAS antibody (Abcam, Cambridge, United Kingdom). The stained sections were scanned and visualized using PANNORAMIC MIDI II and CaseViewer software (3D HISTECH, Budapest, Hungary).

### 2.11 Transcriptome sequencing

Total cellular RNA was prepared by TRIzol (Invitrogen, CA, United States) for transcriptome sequencing. The total RNA quantity and purity were analyzed using Bioanalyzer 2100 and RNA 6000 Nano LabChip Kit (Agilent, CA, United States), respectively; high-quality RNA samples with RIN number >7.0 were used to construct the sequencing library. Then, mRNA was purified from the total RNA (5ug) using Dynabeads Oligo (dT) (Thermo Fisher, CA, United States). During library preparation, RNA is fragmented to ∼300 bp using divalent cations at 94 °C, followed by first-strand cDNA synthesis using SuperScript™ II Reverse Transcriptase (Invitrogen, CA, United States) and second-strand synthesis using DNA Polymerase I (NEB, MA, United States). An A-base was then added to the blunt ends of each strand, and dual-index adapters were ligated to the fragments. Size selection was performed using AMPureXP beads. The libraries are amplified through eight cycles of PCR. The average insert size for the final cDNA libraries was 300 ± 50 bp. Finally, the libraries were sequenced using 2 × 150 bp paired-end reads (PE150) on the Illumina Novaseq™ 6000 platform by LC Bio Technology Co., Ltd. (Hangzhou, China). The bioinformatics pipeline includes the following: adapter trimming and quality filtering using Cutadapt, raw data quality control using FastQC (Q20, Q30 and GC-content), alignment to reference genomes with HISAT2, gene expression quantification using StringTie and ballgown, differential expression analysis with DESeq2 (|log2FC| ≥ 1, FDR <0.05), principal component analysis (PCA) using R, and functional enrichment analysis through KEGG/GO pathways. Other bioinformatic analysis was performed using the OmicStudio tools at https://www.omicstudio.cn/tool.

### 2.12 Dataset

To confirm the 2′-O-methylational modification of SNORD13H on mRNA, we downloaded a gene database of published articles from the Gene Expression Omnibus (GEO) database (GSE77027, https://ncbi.nlm.nih.gov/geo/query/acc.cgi?acc=GSE770 27). It is a CLIP-seq dataset which contains RNA in complex with the 2′-O-methylase FBL (Incarnato et al., 2017).

### 2.13 Statistical analysis

All data were based on at least three independent experiments. Each independent experiment was in triplicates. Data were analyzed using Student’s t-test and ANOVA analysis, which were implemented using GraphPad Prism 9.0 statistical software (CA, United States). Statistically significant differences were deemed only if p < 0.05.

## 3 Results

### 3.1 SNORD13H is downregulated in HCC

**FIGURE 1:**
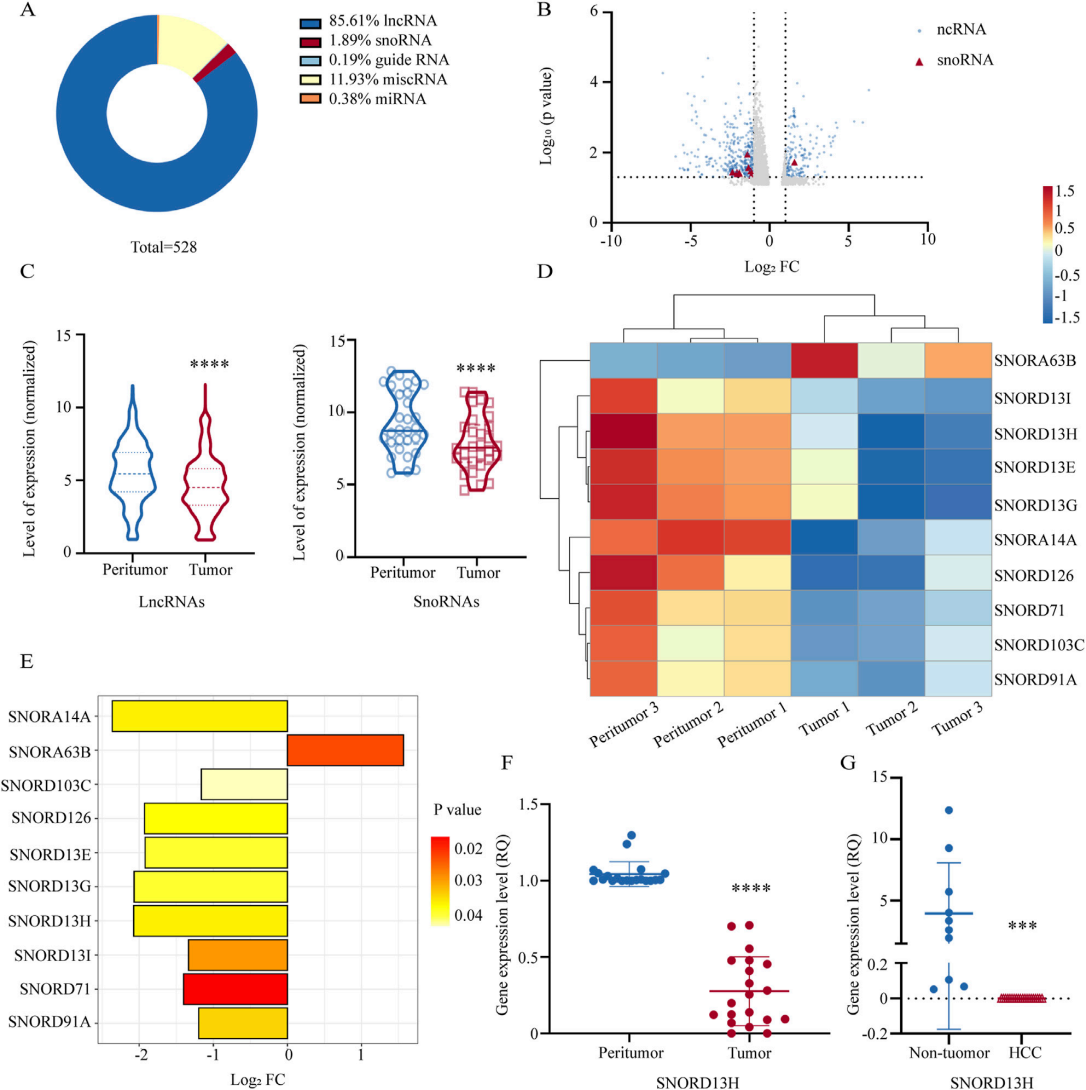
SNORD13H is downregulated in HCC. **(A)** Proportional distribution of differentially expressed ncRNAs in tumor tissues vs. peritumor tissues from HCC patients (three paired samples, p < 0.05, |log2FC|>1). SnoRNAs accounted for 1.89% of all dysregulated ncRNAs (total n = 528). **(B)** Volcano plot of ncRNA expression profiles. Red triangles: differentially expressed snoRNAs (p < 0.05); blue dots: other significant ncRNAs; gray dots: nonsignificant ncRNAs. **(C)** Violin plots of expression levels for differentially expressed lncRNAs (left) and snoRNAs (right) with quartile ranges (dotted/solid lines). Statistical significance was calculated using Student’s t-test (****, p < 0.0001). **(D)** Heatmap of snoRNA expression patterns across matched tumor and peritumor samples. Rows: samples; columns: individual snoRNAs. **(E)** Bar plot of significantly altered snoRNAs (|log2FC|>1.0, p < 0.05). Nine snoRNAs were downregulated and one upregulated. Abscissa: log2FC, ordinate: snoRNAs; bar color indicates the p-value. **(F)** q-PCR validation of SNORD13H suppression in 20 pairs of human HCC tissues and peritumor tissues. Each dot represents one sample. Data were displayed as relative quantification to peritumor tissues. Bars represent mean ± SD. Statistical significance was determined using a two-tailed Student’s t-test (****, p < 0.0001). **(G)** Plasma SNORD13H levels in HCC patients (n = 20) vs. non-tumor controls (n = 10). Bars represent mean ± SD. Relative quantitation was used. Statistical significance was determined using a two-tailed Student’s t-test (***, p = 0.0002).

To identify snoRNAs associated with HCC, we performed RNA sequencing on a cDNA library derived from three pairs of human HCC tissues and matched peritumor tissues. A profile of RNA-sequencing results is displayed (Figure 1A). Using Entrez Gene IDs, we categorized differentially expressed ncRNAs (p < 0.05, Log2 [Fold Change (FC)]>1), with particular focus on lncRNA and snoRNA. We generated a volcano plot highlighting snoRNAs (Figure 1B) and violin plots illustrating overall expression changes in snoRNA and lncRNA (Figure 1C). Notably, snoRNA expression exhibited a significant downward trend in HCC tissues. A heatmap was used for further analysis (Figure 1D). Among these snoRNAs, a C/D box snoRNA, SNORD13H, showed the most pronounced fold changes (Figure 1E). We validated its expression in 20 additional HCC tissue pairs using Q-PCR, confirming significant downregulation in tumors compared to peritumor tissues (Figure 1F). Similarly, SNORD13H levels were reduced in plasma samples from HCC patients relative to non-tumor controls (Figure 1G). Given these findings, we proceeded to investigate the functional roles of SNORD13H in HCC development and progression.

### 3.2 SNORD13H suppresses HCC cell growth and proliferation

**FIGURE 2:**
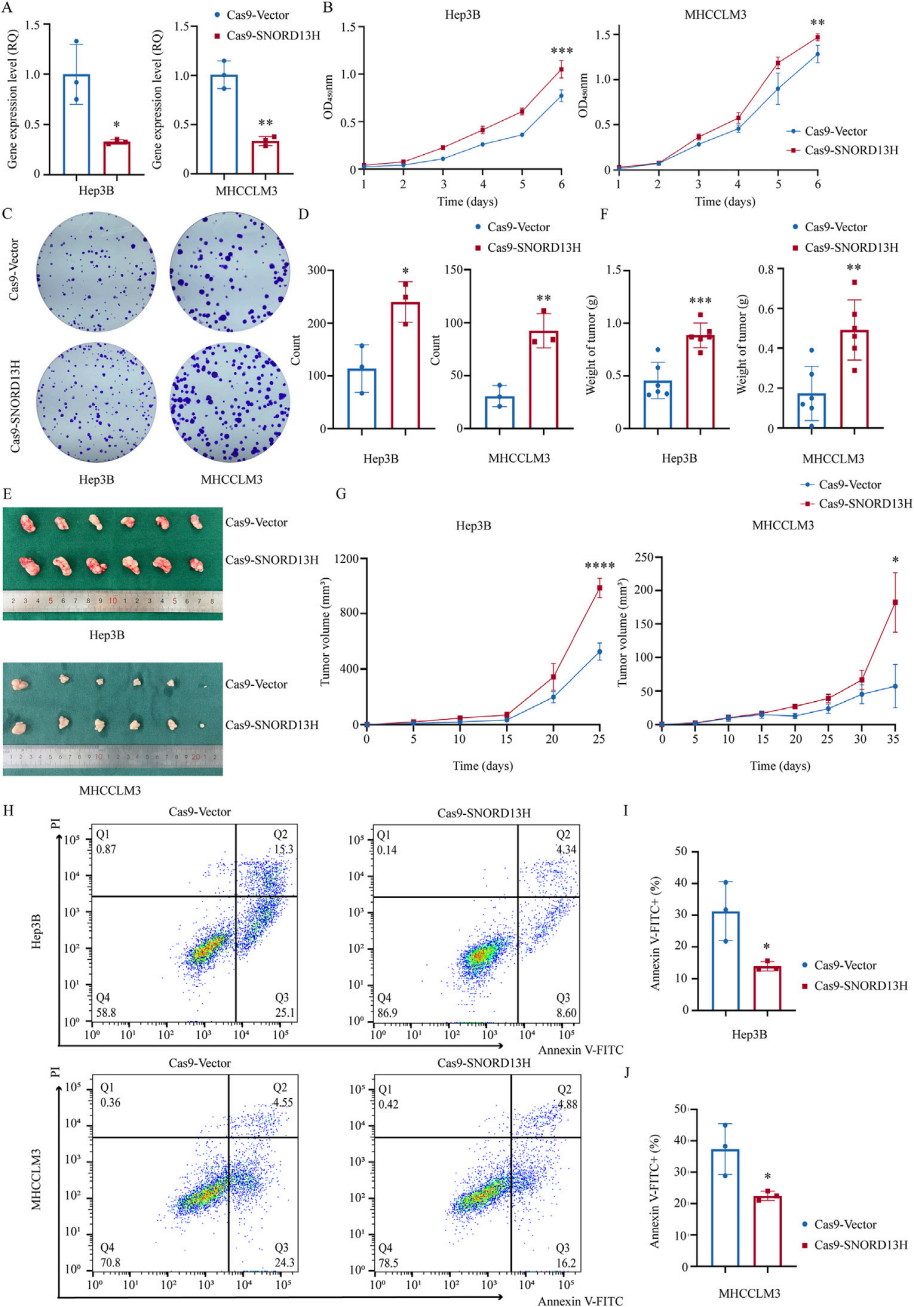
Loss of SNORD13H promotes HCC cell proliferation *in vitro* and *in vivo*. **(A)** Validation of SNORD13H knockout efficiency in Hep3B (left, p = 0.0178) and MHCCLM3 (right, p = 0.0014) cells using q-PCR. Data normalized to vector control. Bars: mean ± SD. Statistical significance was determined using a two-tailed Student’s t-test. **(B)** Cellular growth and proliferation using CCK-8 assay. SNORD13H-knockout cells showed increased absorbance (450 nm) in Hep3B (left, ***, p = 0.0003) and MHCCLM3 (right, **, p = 0.0036). Statistical significance by two-way ANOVA (time × genotype) with t-test at endpoint; dots and bars: mean ± SD. **(C,D)** Colony formation assay **(C)** and quantifications **(D)**. Results were quantified using ImageJ software **(D)**. SNORD13H-knockout cells formed more colonies in Hep3B (*, p = 0.0209) and MHCCLM3 (**, p = 0.0050). **(D)** Each dot represents one independent experiment with bars depicting mean ± SD. Statistical significance was determined using Student’s t-test. **(E–G)** Subcutaneous tumor xenograft mouse models (n = 6 per group). **(E)** Representative tumors at endpoint. **(F)** Tumor weights: Hep3B (left, ***, p = 0.0005) and MHCCLM3 (right, **, p = 0.0032). **(G)** Growth curves (****, p < 0.0001 for Hep3B; *, p = 0.0427 for MHCCLM3). Bars/points: mean ± SD. Statistical significance was calculated using two-way ANOVA and Student’s t-test. **(H–J)** Apoptosis suppression by SNORD13H knockout. **(H)** Flow cytometry plots (Annexin-V FITC/PI staining). **(I,J)** Quantification of apoptotic cells (Annexin-V FITC+): Hep3B (I, *, p = 0.0327) and MHCCLM3 (J, *, p = 0.0353). Data were processed using FlowJo software. Dots: replicates; bars: mean ± SD. Student’s t-test was performed for statistical significance.

We first evaluated SNORD13H expression across seven HCC cell lines and immortalized human hepatocyte cell line L-02 (Supplementary Figure 1A). Hep3B and MHCCLM3 cells were selected for further studies. To mimic the low SNORD13H conditions observed in HCC, we constructed stable SNORD13H-knockout cell lines using the CRISPR/Cas9 system, with Q-PCR confirming knockout efficiency (Figure 2A). Functional assays revealed that SNORD13H-knockout cells displayed significantly increased viability, proliferation, and colony formation capability compared to controls (Figures 2B–D). Conversely, SNORD13H overexpression (Supplementary Figure 1E) suppressed these malignant phenotypes (Supplementary Figures 1F–H). The results support a tumor-suppressive role for SNORD13H *in vitro*.

To validate these findings *in vivo*, we subcutaneously implanted SNORD13H-knockout or control Hep3B/MHCCLM3 cells into BALB/c nude mice, establishing xenograft models. Tumors derived from SNORD13H-knockout cells exhibited larger volumes (Figure 2E), faster growth rates (Figure 2G), and heavier weights (Figure 2F). These results demonstrate that SNORD13H loss enhances development and progression of HCC *in vivo*.

In the exploration on the mechanistic basis of SNORD13H-regulated tumor progression, SNORD13H-knockout cells showed significant resistance to starvation-induced apoptosis (Figures 2H–J). This result could correlate with reduced cleaved Caspase-3 levels (Supplementary Figure 1C), suggesting that SNORD13H modulates apoptosis via Caspase-3 activation. These findings position SNORD13H as a tumor suppressor in HCC that constrains malignant progression through the dual regulation of proliferation and apoptosis.

### 3.3 Decreased SNORD13H promotes global protein translation via 2′-O-methylation of 18S rRNA

**FIGURE 3:**
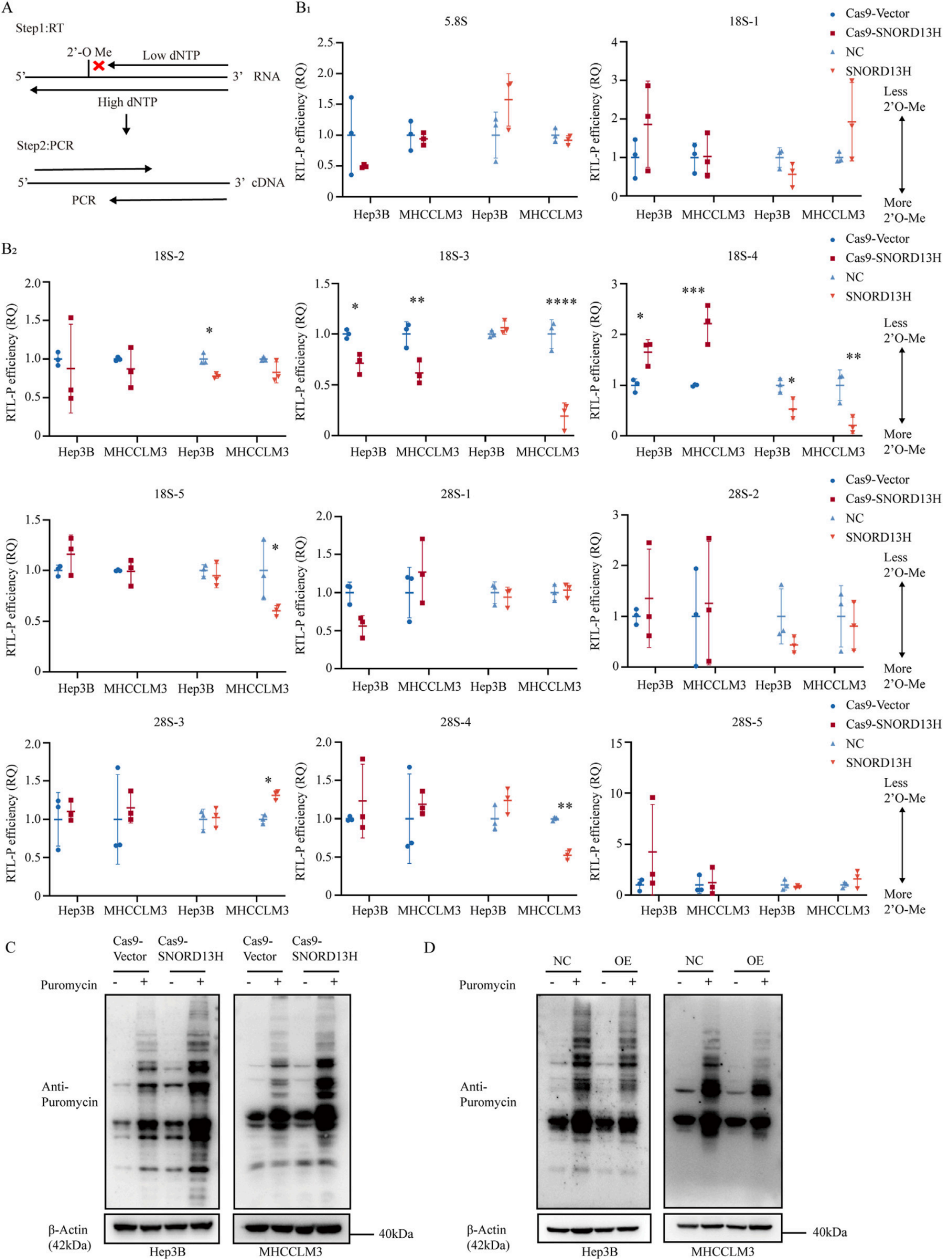
SNORD13H deficiency enhances translational efficiency with reduced 2′-O-methylation of 18S rRNA. **(A)** Schematic representation of the RTL-P assay to quantify RNA 2′-O-methylation. Low dNTP conditions preferentially stall reverse transcription at methylated sites, generating truncated cDNA products detectable through PCR. **(B)** (with subsections B1 and B2) RTL-P assay of rRNA 2′-O-methylation in SNORD13H-knockout cells. Data were measured using ImageJ software. Methylation levels at specific sites in 5.8S, 18S, and 28S rRNAs were normalized to vector controls. Downstream amplicons (Fd) served as the loading control. Each symbol represents one independent experiment. Bars: mean ± SD. Statistical significance was calculated using Student’s t-test (*, p < 0.05; **, p < 0.01; ***, p < 0.001; and ****, p < 0.0001). **(C,D)** Translational profiling through SUnSET assay. SNORD13H-knockout cells **(C)** showed increased puromycin incorporation, indicating elevated global translation, whereas overexpression **(D)** suppressed it.

SnoRNAs primarily function to guide RNA modifications, with rRNA as their main target. As a C/D box snoRNA, it is anticipated that SNORD13H regulates 2′-O-methylation of rRNA, a conserved RNA modification critical for ribosome function. Using RTL-P (Figure 3A), we assessed 2′-O-methylation levels across 18S and 28S rRNA segments in SNORD13H-knockout and overexpressing HCC cells (Supplementary Figures 2, 3), which is referred to previous studies (Dong et al., 2012). Normalized quantification revealed that SNOD13H depletion specifically reduced 2′-O-methylation at the 18S-4 segment, whereas SNORD13H overexpression increased it (Figure 3B).

C/D box snoRNAs are characterized by conserved C box (UGAUGA) and D box (CUGA) motifs. Between these motifs lies the antisense element (ASE), a 10–21 nucleotide guide sequence that base pairs with target RNA to position the modification site and directs 2′-O-methylation (Bratkovic et al., 2020). Unlike canonical Watson–Crick pairing, RNA–RNA interactions in this process are more flexible, permitting noncanonical base pairs. Through comparative analysis, Um1288 was predicted as the most probable SNORD13H-dependent methylation site on 18S rRNA (Supplementary Figure 4C). Negative controls—including known 2′-O-methylate sites guided by other snoRNAs on 18S or 28S rRNA (Dong et al., 2012; Wu et al., 2020; Zhou et al., 2017)—showed no changes in SNORD13H-knockout cells (Supplementary Figure 4A,B). These complementary findings demonstrate a direct mechanistic link between SNORD13H loss and impaired rRNA modification.

Given the role of 2′-O-methylation in rRNA processing and cleavage (Ni et al., 1997), we evaluated its impact on 18S rRNA mutation (Supplementary Figure 4D). SNORD13H knockout decreased the mature-to-precursor ratio of 18S rRNA, whereas overexpression had the opposite effect (Supplementary Figure 4E). No such changes were observed for 28S rRNA, suggesting that SNORD13H specially regulates 18S rRNA maturation via 2′-O-methylation.

Posttranscriptional rRNA modifications play a direct role in regulating ribosome biogenesis and transcript stability (Liu et al., 2019). As ribosomes are vital organelles for protein synthesis (Ahmed et al., 2019), 2′-O-methylation, one of the most abundant rRNA modifications (Gribling-Burrer et al., 2019), has been shown to modulate the ribosome translation efficiency (Yi et al., 2021). The SUnSET assay (Yi et al., 2021; Schmidt et al., 2009) revealed that SNORD13H knockout enhanced the global translation efficiency, whereas overexpression suppressed it (Figures 3C,D). This aligns with its tumor-suppressive role, as elevated translation promotes the proliferation and growth in HCC (Wang B. et al., 2022). Together, these findings demonstrate that SNORD13H loss drives HCC progression by reducing 18S rRNA methylation and increasing protein synthesis.

### 3.4 SNORD13H deficiency activates MAPK/ERK signaling via RAS upregulation in HCC

Although snoRNAs are best known for rRNA modifications, emerging evidence suggests broader regulatory functions. C/D box snoRNAs can bind mRNAs directly, influencing their splicing (Falaleeva et al., 2016; Huang et al., 2017), stability, and protein expression (Wu et al., 2020; Baldini et al., 2022). These promoted us to investigate whether SNORD13H regulates HCC progression through mechanisms beyond rRNA 2′-O-methylation.

**FIGURE 4:**
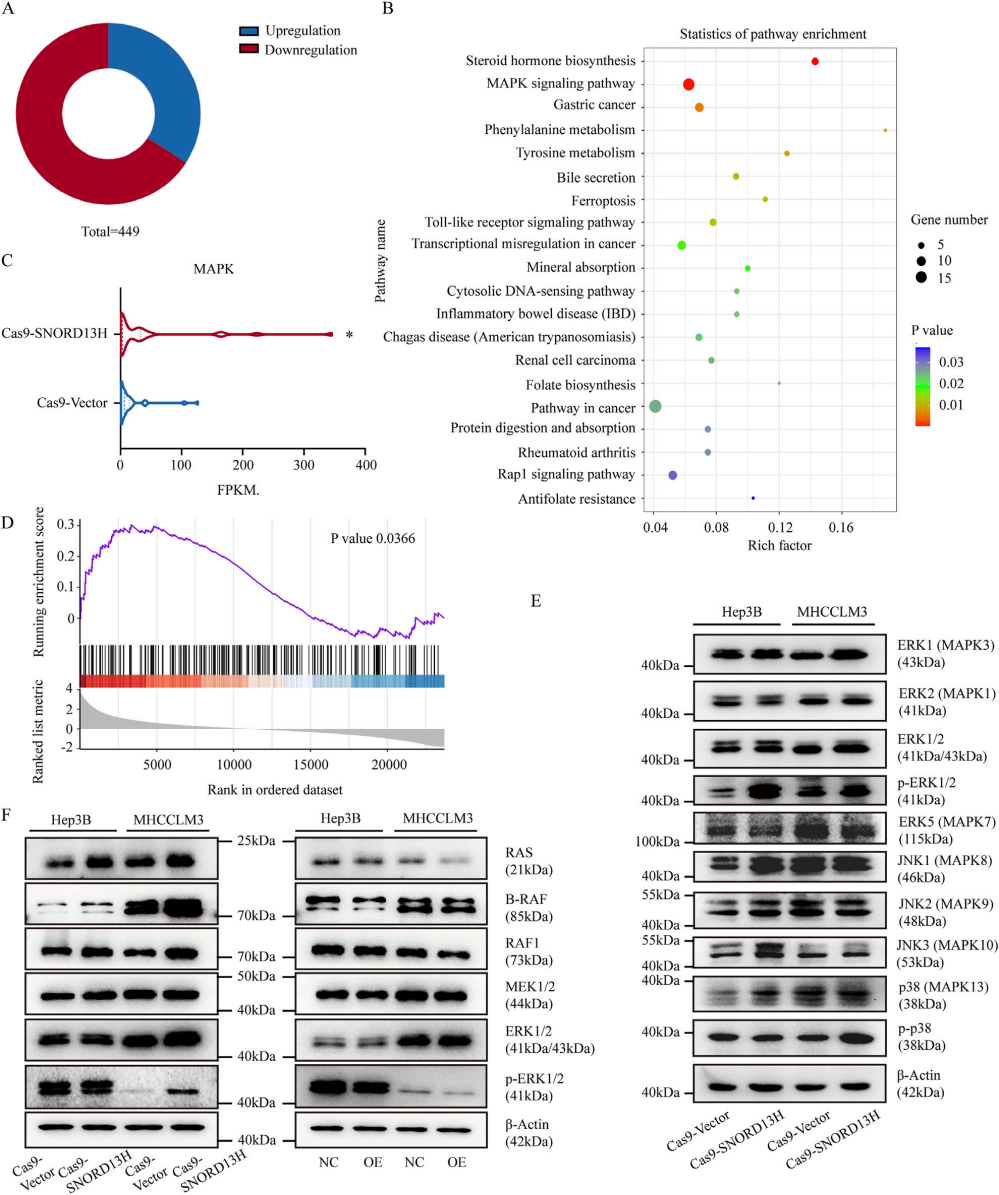
SNORD13H loss activates MAPK signaling through RAS protein. **(A)** Transcriptomic profiling of SNORD13H-knockout cells by RNA sequencing (RNA-seq). Pie chart shows significantly dysregulated genes (p < 0.05, |log2FC|>1; total n = 449). **(B)** KEGG pathway analysis of enriched signaling pathways. Dot size reflects gene counts; color indicates statistical significance (p < 0.05). **(C)** MAPK component expression from RNA-seq. Wilcoxon test confirmed pathway-wide upregulation (*, p = 0.0358). **(D)** GSEA validation of MAPK activation. Normalized enrichment score (NES) = 1.30. **(E)** Western blot analysis of MAPK effectors in SNORD13H-knockout cells containing total/phosphorylated MAPK proteins (ERK1/2, ERK5, p38, and JNK) vs. β-actin control. **(F)** Western blot analysis of RAS/ERK cascade kinases (RAS, BRAF, MEK1/2, RAF1, and ERK1/2). RAS is consistently upregulated in knockout but suppressed in SNORD13H-overexpressing cells.

RNA-seq analysis of SNORD13H-knockout MHCCLM3 cells revealed significant transcriptomic alterations (Figure 4A). The principal component analysis (PCA) of SNORD13H-knockdown vs. control HCC cells reveals distinct transcriptional programs, with PC1 separating by genotypes and PC2 reflecting cell line-specific signatures (Supplementary Figure 5A). KEGG pathway enrichment highlighted the MAPK signaling pathway as the most affected one (Figure 4B), with upregulated mRNA levels of enriched genes (Figure 4C). Gene Set Enrichment Analysis (GSEA) further confirmed MAPK pathway activation in knockout cells (Figure 4D).

To further characterize MAPK pathway activation, we also analyzed RNA-seq data for different expressions of MAPK-associated genes (Supplementary Figures 5C,D) and validated its consistent mRNA changes in selected conditions (Supplementary Figure 5E). Based on the GO enrichment analysis (FDR <0.05) highlighting significant terms across biological processes, molecular functions, and cellular components (Supplementary Figure 5B), we prioritized one gene as a candidate regulator. However, despite transcriptional upregulation of the filtered gene, its corresponding protein levels did not display significant alternations (Supplementary Figure 5F). This discrepancy suggests that SNORD13H regulates MAPK signaling primarily through other modalities.

SNORD13H knockout specially increased phosphorylated ERK1/2 (p-ERK1/2) levels (Figure 4E), indicating ERK1/2 pathway activation instead of MAPK/p38, MAPK/ERK5 and MAPK/JNK1/2/3. Subsequent profiling of MAPK cascade components revealed that RAS protein levels were elevated in SNORD13H-knockout cells but reduced in SNORD13H-overexpressing cells (Figure 4F). Consistent with this, xenograft tumors derived from SNORD13H-knockout cells exhibited higher Ki67 and RAS expressions (Supplementary Figures 6, 7), corroborating enhanced proliferation and MAPK signaling *in vivo*. Our data demonstrate that SNORD13H downregulation in HCC promotes RAS accumulation, leading to constitutive MAPK/ERK pathway activation and driving tumor progression. This expands the functional repertoire of SNORD13H to include direct modulation of oncogenic signaling pathways.

### 3.5 SNORD13H suppresses HCC progression by RAS-mediated cellular activities

The RAS family of small GTPases (including HRAS, KRAS, and NRAS) plays pivotal roles in tumor progression (Akula et al., 2016). To make sure RAS is a key effector of SNORD13H depletion, we generated SNORD13H/RAS double-knockout HCC cells (Supplementary Figure 8A). Compared to SNORD13H-knockout alone, double-knockout cells exhibited rescued viability, impaired colony formation, and increased apoptosis (Supplementary Figures 8B–F, 9). These results indicate that RAS is essential for the tumor progression promotion induced by SNORD13H loss.

**FIGURE 5:**
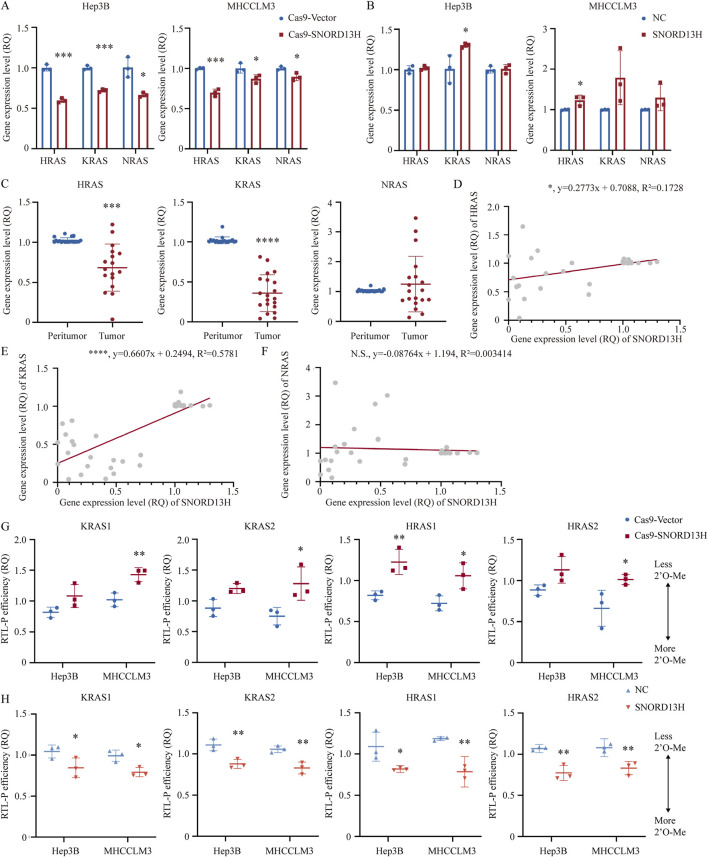
SNORD13H regulates RAS expression through mRNA 2′-O-methylation. **(A–C)** Q-PCR analysis of RAS isoforms. **(A)** SNORD13H-knockout cells showed reduced HRAS and KRAS mRNAs vs. control. **(B)** SNORD13H-overexpression cells exhibited increased HRAS and KRAS mRNAs. **(C)** HRAS and KRAS mRNAs were downregulated in HCC tumors (n = 20) vs. peritumor tissues. Bars: mean ± SD (Student’s t-test) (*, p < 0.05; **, p < 0.01; ***, p < 0.001; and ****, p < 0.0001). **(D–F)** Expression correlation analysis. **(D,E)** SNORD13H expression positively correlated with HRAS (D, *, p = 0.0117) and KRAS (E, ****, p < 0.0001) mRNAs in human tissue samples (Pearson correlation analysis). **(F)** No correlation with NRAS (n.s., p = 0.7239). Gray dots: individual samples; red line: linear regression. **(G,H)** RTL-P assays of RAS mRNA 2′-O-methylation. **(G)** SNORD13H knockout reduced 2′-O-methylation. **(H)** SNORD13H overexpression increased 2′-O-methylation. Fd bands: loading controls; normalization was applied with control groups (Cas9-Vector/NC cells). Each symbol denotes one independent experiment and bars display mean ± SD. Statistical significance was determined using a two-tail Student’s t-test (*, p < 0.05; **, p < 0.01; ***, p < 0.001; and ****, p < 0.0001).

When we explored reasons for altered RAS expression, we found that HRAS and KRAS mRNA levels were paradoxically reduced (Figures 5A,C) despite elevated RAS protein levels in SNORD13H-knockout cells. Conversely, SNORD13H overexpression increased HRAS and KRAS mRNA levels (Figure 5B), with positive correlations observed in HCC tissues (Figures 5D–F). This inverse relationship between RAS mRNA and protein levels suggested post-transcriptional regulation.

Certain ncRNAs directly bind mRNAs to modulate gene expression through mechanisms, including translation inhibition and transcript stabilization (Latonen et al., 2018; He and Hannon, 2004). mRNA 2′-O-methylation guided by snoRNAs shares the same functions (Elliott et al., 2019). These findings prompted us to investigate whether SNORD13H regulates RAS mRNA similarly through direct 2′-O-methylation.

Given that FBL is the specific methyltransferase combining with snoRNAs, analysis of FBL CLIP-seq data (GSE77027) separately found two KRAS and HRAS mRNA coding regions. Primers for RTL-P were designed focusing on these four FBL-binding regions. Assays confirmed reduced 2′-O-methylation of RAS mRNA in SNORD13H-knockout cells (Figure 5G; Supplementary Figure 10A) and enhanced methylation upon SNORD13H overexpression (Figure 5H; Supplementary Figure 10B). Meanwhile, FBL protein levels were not affected by SNORD13H/RAS knockout (Supplementary Figure 10C). These revealed RAS mRNA 2′-O-methylation induced by SNORD13H. We also predicted SNORD13H binding sites within KRAS and HRAS mRNAs (Supplementary Figure 10D). Notably, 2′-O-methylation suppresses translation (Elliott et al., 2019), perhaps explaining why RAS protein levels increased despite mRNA downregulation in SNORD13H-deficient cells. Our findings establish a novel mechanism, whereby SNORD13H inhibits HCC progression by guiding 2′-O-methylation of RAS mRNA. Under this condition, RAS protein expression and MAPK/ERK pathway activation are promoted by SNORD13H loss. This dual regulatory role underscores SNORD13H’s tumor-suppressive function in HCC.

### 3.6 SNORD13H regulates HCC progression via FBL-dependent 2′-O-methylation of RAS mRNA

**FIGURE 6:**
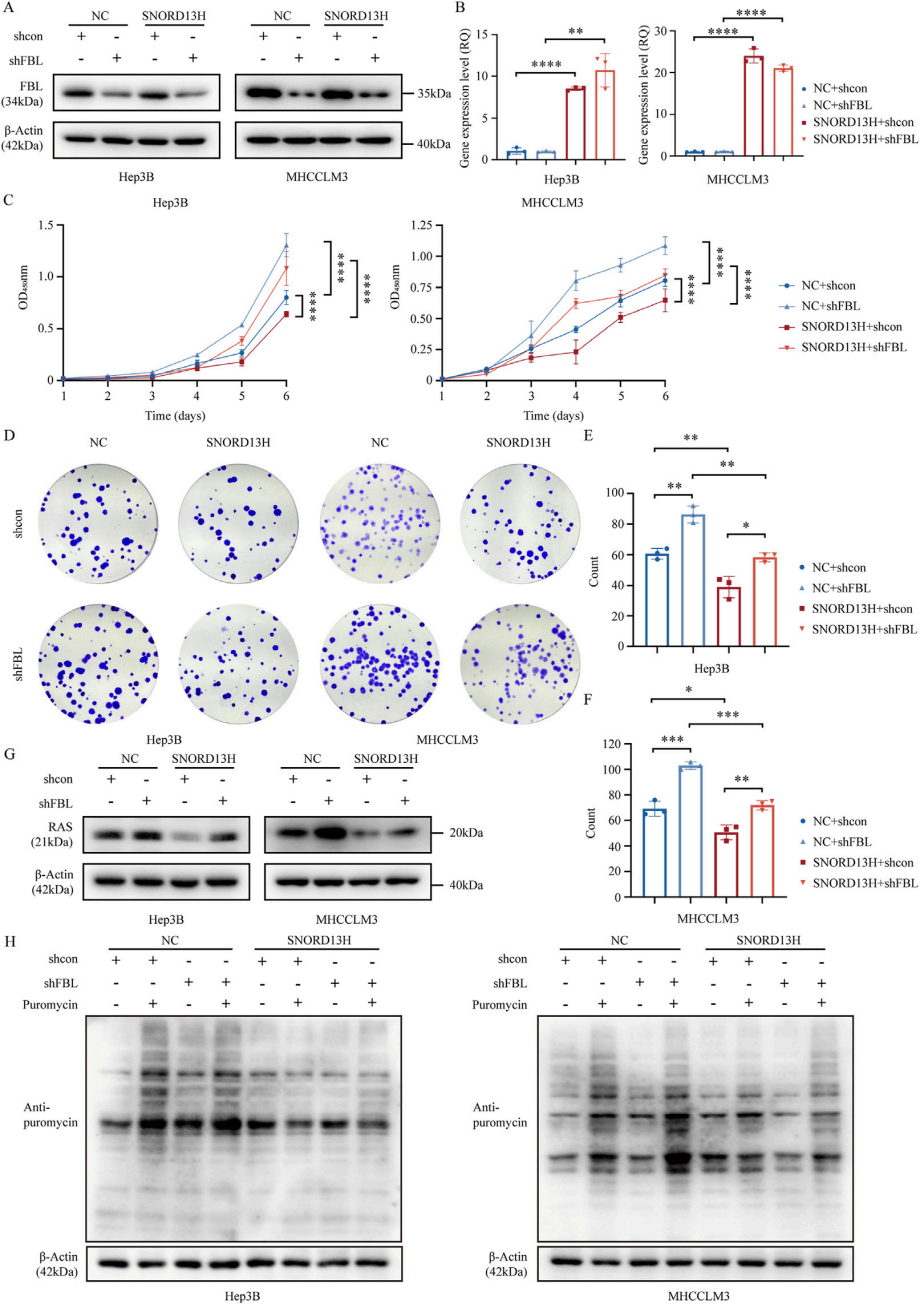
FBL mediates the tumor-suppressive effects of SNORD13H. **(A)** Validation of FBL knockout in SNORD13H-overexpressing HCC cells using Western blot. **(B)** q-PCR validation of SNORD13H overexpression stability after FBL knockout. Data were relatively quantified to control cells. Bars: mean ± SD (Student’s t-test) (*, p < 0.05; **, p < 0.01; ***, p < 0.001; and ****, p < 0.0001). **(C)** Proliferation assay through CCK-8. FBL depletion showed restored growth and proliferation in SNORD13H-overexpressing cells vs. shcon. Ordinate was the absorbance at wavelength of 450 nm. Statistical significance was analyzed using two-way ANOVA and a two-tail t-test. Symbols and bars indicate mean ± SD (*, p < 0.05; **, p < 0.01; ***, p < 0.001; and ****, p < 0.0001). **(D–F)** Clonogenic potential analysis. **(D)** Representative images of colony formation assays. **(E,F)** Quantification revealed that FBL knockout reversed SNORD13H-overexpression-induced suppression (t-test). Data were processed using ImageJ software. **(E)** Hep3B; **(F)** MHCCLM3. Dots: replicates; bars: mean ± SD (Student’s t-test) (*, p < 0.05; **, p < 0.01; ***, p < 0.001; and ****, p < 0.0001). **(G)** Western blot showed that FBL knockout increased RAS levels in SNORD13H-overexpressing cells (vs. β-actin loading control). **(H)** Translational recovery. SUnSET assays demonstrated elevated puromycin incorporation in FBL-depleted SNORD13H-overexpressing cells, indicating restored translation.

FBL, the catalytic component of snoRNPs, is indispensable for snoRNA-guided 2′-O-methylation. To test whether FBL mediates SNORD13H’s effects, we depleted FBL in SNORD13H-overexpressing cells (Figure 6A). Moreover, overexpression efficiency of SNORD13H remained unchanged (Figure 6B). Whereas SNORD13H overexpression alone suppressed HCC cell growth, stemness, and proliferation, FBL knockout reversed these effects (Figures 6C–F). Consistent with this, FBL depletion restored RAS protein levels (Figure 6G) and global translational efficiency (Figure 6H), proving that SNORD13H’s tumor-suppressive function requires FBL-dependent methylation.

**FIGURE 7:**
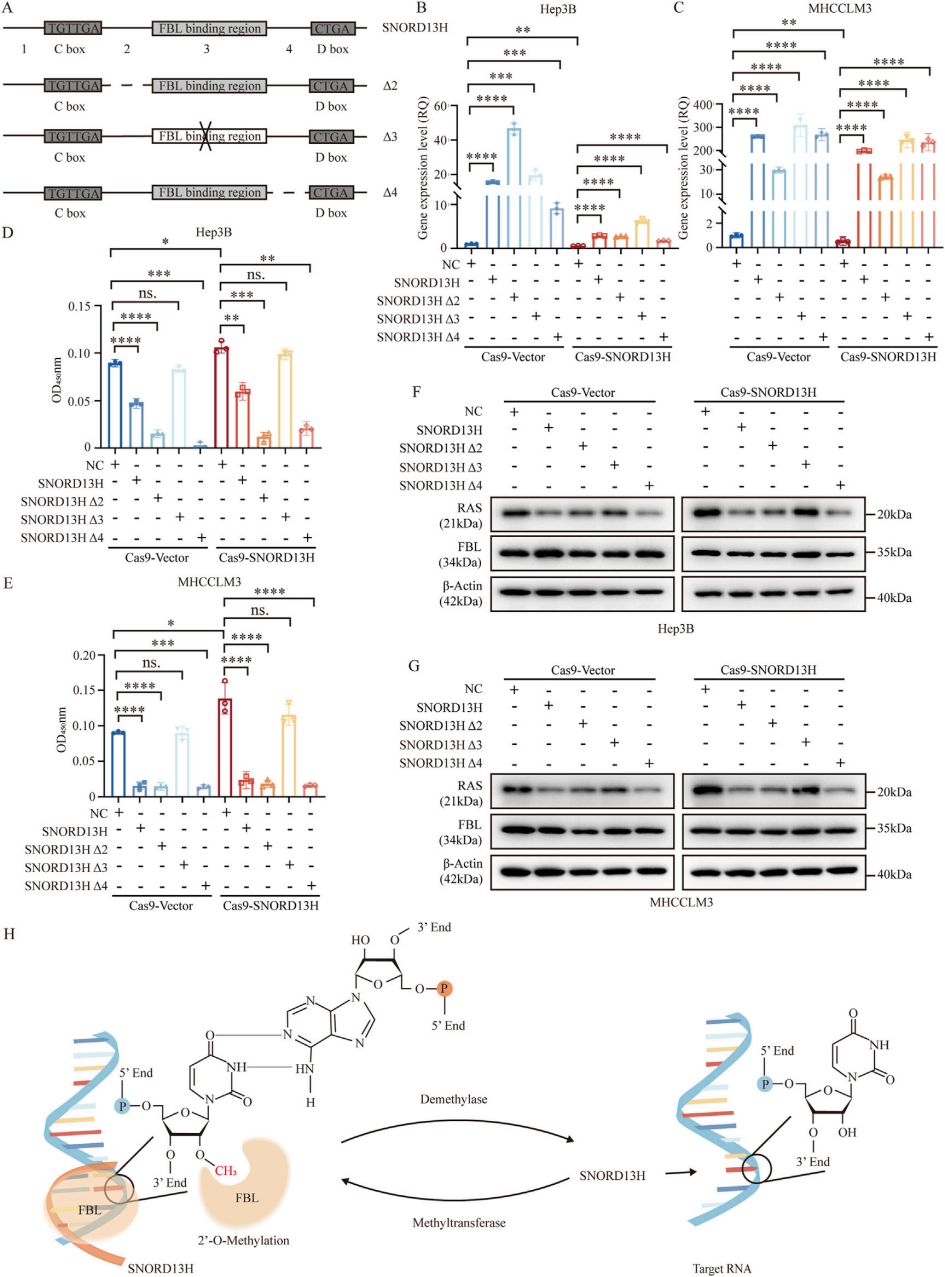
SNORD13H regulates HCC progression through site-specific 2′-O-methylation. **(A)** Design of SNORD13H mutants. Schematic representation of antisense sequence mutations (dotted lines or “ × ”) targeting predicted functional domains. Wild-type sequences are shown as solid lines. **(B,C)** Validation of mutant expression. q-PCR confirmed successful transfection of SNORD13H mutants in SNORD13H-knockout cells. **(B)** Hep3B; **(C)** MHCCLM3. Data normalized to vector controls (NC). Bars: mean ± SD. Statistical significance was calculated using Student’s t-test (*, p < 0.05; **, p < 0.01; ***, p < 0.001; and ****, p < 0.0001). **(D,E)** Proliferation rescue assays. CCK-8 plots show differential growth restoration by mutants in **(D)** Hep3B and **(E)** MHCCLM3 cells (vs. vector controls). Results were displayed as the absorbance at wavelength of 450 nm. Statistical significance was measured using a two-tail t-test (*, p < 0.05; **, p < 0.01; ***, p < 0.001; and ****, p < 0.0001). **(F,G)** RAS protein modulation. Western blots demonstrate mutant-specific effects on RAS levels in **(F)** Hep3B and **(G)** MHCCLM3. β-actin loading control. **(H)** Mechanistic model. SNORD13H guides FBL to catalyze 2′-O-methylation at specific rRNA/mRNA sites through antisense complementarity.

Based on CLIP-seq data (GSE77027) and structural predictions, we divided SNORD13H into four fragments (Figure 7A) and generated three mutants (mutation of segment 2, segment 3, and segment 4, named Δ2, Δ3, and Δ4, respectively) using their antisense sequences (Supplementary Table 1). Notably, segment 3 contained FBL-binding sequences. These mutants were transfected into SNORD13H-knockout cells (Figures 7B,C). In addition, it revealed that Δ3 most weakly rescued cell viability and proliferation (Figures 7D,E). Moreover, Δ3 minimally affected RAS protein levels (Figures 7F,G). These indicate that segment 3 is critical for SNORD13H’s ability to 2′-O-methylate and restrain RAS protein levels. To sum up, we confirmed that SNORD13H’s tumor-suppressive effects depend on FBL-mediated 2′-O-methylation on rRNA and RAS mRNA (Figure 7H). Segment 3 of SNORD13H is essential for targeting RAS mRNA. Reduced 2′-O-methylation of RAS mRNA in SNORD13H-deficient cells drives tumor promotion and progression.

## 4 Discussion

HCC is characterized by high mortality and poor prognosis, largely due to late-stage diagnosis and its high metastatic potential, which limit therapeutic efficacy. Although early detection is critical for improving outcomes, current diagnostic methods lack sufficient accuracy and efficiency (Wilson et al., 2017; Miryam Müller et al., 2020; Zhang et al., 2021). Prior to 2017, a comprehensive study examined the expression and clinical significance of snoRNAs across approximately 31 cancer types, highlighting the significant roles of snoRNAs as tumor markers and therapeutic targets (Gong et al., 2017). In this study, we further explore the potential of SNORD13H as a biomarker for tumor screening, highlighting its reduced expression in the peripheral plasma of HCC patients. These findings underscore the primary clinical relevance of our research.

Subsequent investigations revealed that reduced SNORD13H levels diminish 2′-O-methylation of 18S rRNA, thereby enhancing global translation efficiency. Although individual rRNA modifications are often considered to exert limited effects on ribosome function, it is generally accepted that cumulative alterations in rRNA modifications collectively influence ribosomal activity (Liang et al., 2007; Baudin-Baillieu et al., 2009). Nevertheless, emerging evidence shows that changes in individual snoRNAs can affect ribosome stability, protein translation, and cellular phenotypes. For instance, SNORA18L5-mediated pseudouridylation improves rRNA maturation and reduces ribosomal stress (Cao et al., 2018), and SNORD11B modifies 18S rRNA at G509 to boost protein synthesis (Bian et al., 2023). Consequently, it is plausible that reduced SNORD13H levels enhance ribosomal translation. This increase in global protein synthesis may contribute to augmented cellular growth, proliferation, and HCC progression.

Through comprehensive investigation, we further identified that decreased SNORD13H levels lead to MAPK pathway activation, as revealed by RNA-seq analysis. The MAPK signaling pathway, a prototypical signaling cascade, is intricately linked to essential cellular processes, including proliferation, differentiation, and survival. Furthermore, the MAPK/ERK pathway modulates various downstream transcription factors and regulates transcription factors and cytokines, which, in turn, feedback to regulate the MAPK/ERK pathway itself, forming a self-sustaining feedback loop (Liu et al., 2018). It is probable that the MAPK-related genes identified in the RNA-seq results represent downstream factors and reflect the MAPK pathway activation (Supplementary Figures 5C,D). The activated MAPK pathway may also contribute to the upregulation of cyclin D1 and the suppression of apoptosis-related protein activity (Supplementary Figure S1C,D). However, there were no significant differences between SNORD13H-knockout cells and control cells (Supplementary Figure 1B). It is reasonable that numerous regulatory proteins involved in cell cycle progression are profoundly influenced by various other factors (Fang et al., 2017). Subsequent rescue experiments in SNORD13H/RAS double-knockout cells indicated that SNORD13H loss activates the MAPK pathway by increasing RAS protein levels.

In this study, SNORD13H was also found to mediate 2′-O-methylation of RAS mRNA, probably altering the abundance of RAS mRNA and protein levels. Using SNORD13H mutants and FBL-knockout models, we experimentally confirmed that this regulation depends on FBL, the essential and evolutionarily conserved methyltransferase for 2′-O-methylation. Nonetheless, alternative mechanisms by which SNORD13H may influence RAS expression cannot be entirely dismissed. The direct interaction of snoRNAs with proteins offers a straightforward mechanism for regulating protein functions (Chen et al., 2023). Altered rRNA 2′-O-methylation has also been reported to selectively affect the translation of specific proteins without altering their mRNA levels (Wang K. et al., 2022).

Our study establishes SNORD13H as a dual-function regulator in HCC progression beyond its canonical roles in rRNA modification. Key precedents support this. The capacity of snoRNAs to regulate diverse RNA targets has been recognized since Hartshorne’s demonstration that U3 (SNORD3A) interacts with both 5′ETS rRNA and mRNAs (Hartshorne, 1998). Subsequent research has demonstrated that C/D box snoRNAs can modify rRNA and bind mRNA through complementary base pairing to influence splicing and translation efficiency (e.g., SNORD27) (Falaleeva et al., 2016; Huang et al., 2017); many also combine with target mRNA, regulating transcript stability (e.g., SNORD116) (Wu et al., 2020; Baldini et al., 2022). A prime example is U32A, which simultaneously guides rRNA methylation and stabilizes PXDN mRNA to decrease its translation (Elliott et al., 2019). Furthermore, certain snoRNAs utilize the same antisense elements to modify multiple RNA targets (Bian et al., 2023), revealing an unexpected versatility in their regulatory potential. These established paradigms of snoRNA pleiotropy provided the mechanistic foundation for our investigation of SNORD13H’s noncanonical regulation of RAS mRNA in hepatocellular carcinoma, where we demonstrate its ability to coordinate both rRNA processing and oncogenic signaling through distinct RNA interaction modalities.

The primary limitation of the current study is the lack of sequencing evidence regarding 2′-O-methylation and RNA–RNA interaction. Although Chuan H. et al. reported a 2′-O-methylated (Nm)-seq method for Nm sites in human mRNA as early as 2017 (Dai et al., 2017), there is a lack of commercial sequencing methods specific to RNA 2′-O-methylation. Previous research suggests that analogous sequencing methods could serve as a reference on rRNA 2′-O-methylation (Yi et al., 2021). The 2′-O-methylation of mRNA is typically assessed using the RTL-P assay in published studies (Elliott et al., 2019). Recently, the latest research introduced a novel methodology for transcriptome-wide snoRNA target identification, revealing the enhanced translation of specific proteins through the interaction of snoRNA, mRNA, and 7SL RNA (Liu et al., 2024), offering new insights into snoRNA functions.

SNORD13H is present only in the genomes of higher primates and absent in mice, which limits traditional animal experimentation but may suggest an evolutionarily acquired protective role. In this study, we confirmed its potential diagnostic value. With ongoing advances in AAV vector engineering and biomedical technologies, gene delivery and therapeutic applications targeting SNORD13H are becoming increasingly feasible. Therefore, the physiological roles of SNORD13H *in vivo* and its therapeutic potential warrant further comprehensive investigation.

## Data Availability

The raw data of RNA-seq presented in the study are deposited in the SRA repository, accession number PRJNA1304434. Datasets are available on request. The raw data supporting the conclusions of this article will be made available by the authors, without undue reservation.

## Abbreviations

HCC, hepatocellular carcinoma; NSCLC, non-small-cell lung cancer; CRC, colorectal cancer; snoRNA, small nucleolar RNA; ncRNA, noncoding RNA; Nm, 2′-O-methylation; Ψ, pseudouridylation; RTL-P, reverse transcription at low dNTP concentrations followed by PCR; SUnSET, surface sensing of translation; KEGG, Kyoto Encyclopedia of Genes and Genomes; GEO, Gene Expression Omnibus; RNA-seq, RNA sequencing; CLIP-seq, Crosslinking-immunoprecipitation and high-throughput sequencing; MAPK, mitogen-activated protein kinase; FBL, fibrillarin.
