# Supplementary material for: Small nucleolar RNA SNORD13H suppresses tumor progression via FBL-dependent 2′-O-methylation in hepatocellular carcinoma
Source: Front Genet. 2025 Aug 21;16:1620552. doi: 10.3389/fgene.2025.1620552 (PMC12408296; doi:10.3389/fgene.2025.1620552)
Supplement: Supplementary file 1 [file Table1.docx]

Supplementary Tables

# Supplementary Table 1. Sequences for gene editing.

| Name | Sequencing (5’-3’) |
| --- | --- |
| Human SNORD13H sgRNA | 5’-AACACCCAATCAACACACTT-3’ |
| Human HRAS shRNA | 5’-GACGTGCCTGTTGGACATCCT-3’ |
| Human KRAS shRNA | 5’-TTATTTCCTACTAGGACCA-3’ |
| Human NRAS shRNA | 5’-CAGTGCCATGAGAGACCAATA-3’ |
| Human FBL shRNA | 5’-CCTTGAGCCATATGAAAGAGA-3’ |
| Human SNORD13H | 5’-ATCCTTTTGTAGTTCCTAAGTGTGTTGATTG GGTGTTCACGTGCATGTGTGAGATGTGGCACCCTTGAATCTTGTTATGACATCAGTACATTTCCCGTCTGACA-3’ |
| Human SNORD13H Δ2 | 5’-ATCCTTTTGTAGTTCCTAAGTGTGTTGAAA CCCACAAGTGCACGTACACACTCTTGTGGCACCCTTGAATCTTGTTATGACATCAGTACATTTCCCGTCTGACA-3’ |
| Human SNORD13H Δ3 | 5’-ATCCTTTTGTAGTTCCTAAGTGTGTTGATTG GGTGTTCACGTGCATGTGTGAGAACACCGTGGGAACTTAGAACAATACTCATCAGTACATTTCCCGTCTGACA-3’ |
| Human SNORD13H Δ4 | 5’-ATCCTTTTGTAGTTCCTAAGTGTGTTGATTG GGTGTTCACGTGCATGTGTGAGATGTGGCACCCTTGAATCTTGTTATGACTAGTCATGTAAAGGGCACTGACA-3’ |

# Supplementary Table 2. Primers for Q-PCR.

| Name | Sequencing (5’-3’) |
| --- | --- |
| Actin-F | 5’-GTCTTCCCCTCCATCGTG-3’ |
| Actin-R | 5’-AGGTGTGGTGCCAGATTTTC-3’ |
| U6-F | 5’-TGGAACGCTTCACGAATTTGCG-3’ |
| U6-R | 5’-GGAACGATACAGAGAAGATTAGC-3’ |
| SNORD13H-F | 5’-ATCCTTTTGTAGTTCATGAGC-3’ |
| SNORD13H-R | 5’-GGTCAGACGGGTAATGTGCCC-3’ |
| HRAS-F-1 | 5’-ATGACGGAATATAAGCTGGTGGT-3’ |
| HRAS-R-1 | 5’-GGCACGTCTCCCCATCAATG-3’ |
| HRAS-F-2 | 5’-GACGTGCCTGTTGGACATC-3’ |
| HRAS-R-2 | 5’-CTTCACCCGTTTGATCTGCTC-3’ |
| KRAS-F-1 | 5’-ACAGAGAGTGGAGGATGCTTT-3’ |
| KRAS-R-1 | 5’-TTTCACACAGCCAGGAGTCTT-3’ |
| KRAS-F-2 | 5’-GAGTACAGTGCAATGAGGGAC-3’ |
| KRAS-R-2 | 5’-CCTGAGCCTGTTTTGTGTCTAC-3’ |
| NRAS-F-1 | 5’-ATGACTGAGTACAAACTGGTGGT-3’ |
| NRAS-R-1 | 5’-CATGTATTGGTCTCTCATGGCAC-3’ |
| NRAS-F-2 | 5’-CAGGGAGCAGATTAAGCGAGT-3’ |
| NRAS-R-2 | 5’-GGGCTTGTTTTGTATCAACTGTC-3’ |
| Name | Sequencing (5’-3’) |
| EFNA1-F | 5’-TCAGGCCCATGACAATCCAC-3’ |
| EFNA1-R | 5’-GTGACCGATGCTATGTAGAACC-3’ |
| EFNA3-F | 5’-CATGCGGTGTACTGGAACAG-3’ |
| EFNA3-R | 5’-AGATAGTCGTTCACGTTCACCT-3’ |
| DUSP1-F | 5’-AGTACCCCACTCTACGATCAGG-3’ |
| DUSP1-R | 5’-GAAGCGTGATACGCACTGC-3’ |
| MAP2K6-F | 5’-GAAGCATTTGAACAACCTCAGAC-3’ |
| MAP2K6-R | 5’-CCTGGCTATTTACTGTGGCTC-3’ |
| TGFβ-3-F | 5’-ACTTGCACCACCTTGGACTTC-3’ |
| TGFβ-3-R | 5’-GGTCATCACCGTTGGCTCA-3’ |
| TGFI-F | 5’-ATGACCCTCACCTCTATGTACC-3’ |
| TGFI-R | 5’-CACAGTTCACAGTTACAATCCCA-3’ |

# Supplementary Table 3. Primers for RTL-P assay.

| Name | Sequencing (5’-3’) |
| --- | --- |
| 5.8S-Fu | 5’-CGACTCTTAGCGGTGGATCACTCGGCT-3’ |
| 5.8S-Fd | 5’-CAGGACACATTGATCATCGACACTTCGAAC-3’ |
| 5.8S-R | 5’-AAGCGACGCTCAGACAGGCGTAGC-3’ |
| 18S-1-Fu | 5’-AGTACGCACGGCCGGTACAGTGAAAC-3’ |
| 18S-1-Fd | 5’-GGATGCGTGCATTTATCAGATCAAAACC-3’ |
| 18S-1-R | 5’-ATCGAACCCTGATTCCCCGTCACCC-3’ |
| 18S-2-Fu | 5’-CTCGATGCTCTTAGCTGAGTGTCCCGC-3’ |
| 18S-2-Fd | 5’-GAATAGGACCGCGGTTCTATTTTGTTGGT-3’ |
| 18S-2-R | 5’-CATTCTTGGCAAATGCTTTCGCTCTGG-3’ |
| 18S-3-Fu | 5’-CCAGAGCGAAAGCATTTGCCAAGAATG-3’ |
| 18S-3-Fd | 5’-CGAAAGTCGGAGGTTCGAAGACGATCAG-3’ |
| 18S-3-R | 5’-CGGGTGAGGTTTCCCGTGTTGAGTCAAAT-3’ |
| 18S-4-Fu | 5’-TTGACTCAACACGGGAAACCTCACCC-3’ |
| 18S-4-Fd | 5’-TCTGTGATGCCCTTAGATGTCCGGG-3’ |
| 18S-4-R | 5’-TCAACGCAAGCTTATGACCCGCACTTAC-3’ |
| 18S-5-Fu | 5’-GTAAGTGCGGGTCATAAGCTTGCGTTGA-3’ |
| 18S-5-Fd | 5’-CGTCGCTACTACCGATTGGATGGTTTAGTG-3’ |
| 18S-5-R | 5’-GTTCGACCGTCTTCTCAGCGCTCCG-3’ |
| Name | Sequencing (5’-3’) |
| 28S-1-Fu | 5’-GGCACGAGACCGATAGTCAACAAGTACCG-3’ |
| 28S-1-Fd | 5’-CAAGAGGGCGTGAAACCGTTAAGAGGT-3’ |
| 28S-1-R | 5’-ACCCGCCGCCGGGTTGAATCCT-3’ |
| 28S-2-Fu | 5’-TCCTGCTCAGTACGAGAGGAACCGC-3’ |
| 28S-2-Fd | 5’-GGGCGAAGCTACCATCTGTGGGAT-3’ |
| 28S-2-R | 5’-GTTTCCCAGGACGAAGGGCACTCC-3’ |
| 28S-3-Fu | TCCGGTAAAGCGAATGATTAGAGGTCTTG |
| 28S-3-Fd | ATGAACCGAACGCCGGGTTAAGGC |
| 28S-3-R | 5’-TTCAGGGCTAGTTGATTCGGCAGGTG-3’ |
| 28S-4-Fu | 5’-CTGCGGTGAGCCTTGAAGCCTAGGG-3’ |
| 28S-4-Fd | 5’-TTGAACATGGGTCAGTCGGTCCTGAGAG-3’ |
| 28S-4-R | 5’-ATCGGTCGCGTTACCGCACTGGAC-3’ |
| 28S-5-Fu | 5’-TGTAAATCTCGCGCCGGGCCGTA-3’ |
| 28S-5-Fd | 5’-GGATAAGGATTGGCTCTAAGGGCTGGGT-3’ |
| 28S-5-R | 5’-ATCCACGGGAAGGGCCCGGCT-3’ |
| 18S G1490-Fu | 5’-CACCCGAGATTGAGCAATAACA-3’ |
| 18S G1490-Fd | 5’-TACACTGACTGGCTCAGCGTG-3’ |
| 18S G1490-R | 5’-GCTTATGACCCGCACTTACTGG-3’ |
| 18S C1703-Fu | 5’-GCGTTGATTAAGTCCCTGCC-3’ |
| Name | Sequencing (5’-3’) |
| 18S C1703-R | 5’-CTTCTCAGCGCTCCGCC-3’ |
| 28S G3878-Fu | 5’-CCTACTATCCAGCGAAACCAC-3’ |
| 28S G3878-Fd | 5’-AAAGAAGACCCTGTTGAGCTT-3’ |
| 28S G3878-R | 5’-TATTCTACACCTCTCATGTCT-3’ |
| 28S G4593-Fu | 5’-TAATCCTGCTCAGTACGAGA-3’ |
| 28S G4593-Fd | 5’-CTGTATGTGCTTGGCTGAG-3’ |
| 28S G4593-R | 5’-ATTCTGACTTAGAGGCGTTCA-3’ |
| HRAS-1-Fu | 5’-ATGACGGAATATAAGCTGGTGGTG-3’ |
| HRAS-1-Fd | 5’-ACTATAGAGGATTCCTACCGGAAGC-3’ |
| HRAS-1-Re | 5’-GTATCCAGGATGTCCAACAGGC-3’ |
| HRAS-2-Fu | 5’-ATGCCTTCTACACGTTGGTGC-3’ |
| HRAS-2-Fd | 5’-GCTGCATGAGCTGCAAGT-3’ |
| HRAS-2-Re | 5’-TCACCTGCGTCAGGAGA-3’ |
| KRAS-1-Fu | 5’-GGACTCTGAAGATGTACCTATGGTCC-3’ |
| KRAS-1-Fd | 5’-ACAAGACAGAGAGTGGAGGATGC-3’ |
| KRAS-1-Re | 5’-TCTGTATTGTCGGATCTCTCTCACC-3’ |
| KRAS-2-Fu | 5’-CTGGGTGTTGATGATGCCTTCTATAC-3’ |
| KRAS-2-Fd | 5’-CTTAAGGCATACTAGTACAAGTGG-3’ |
| KRAS-2-Re | 5’-AGCTAACAGTCTGCATGGAGC-3’ |

# Supplementary Table 4. Primers for ribosomal RNA processing analysis.

| Name | Sequencing (5’-3’) |
| --- | --- |
| 1 | 5’-GCGGGACACTCAGCTAAGAGC-3’ |
| 2 | 5’-GGCCCTGTAATTGGAATGAG-3’ |
| 3 | 5’-GCGCCCGTCGGCATGTATTAGCTC-3’ |
| 4 | 5’-CTCGCCGCGCTCTACCTTACCTACCTGG-3’ |
| 5 | 5’-GGCAGGATCAACCAGGTAGGTAAGG-3’ |
| 6 | 5’-GGGGGGCGGGTGGTTGGGGCGTCC-3’ |
| a | 5’-CTTACGGTACTTGTTGACTATCGGTCTCG-3’ |
| b | 5’-CCAAGTCCTTCTGATCGAGGCCC-3’ |
| c | 5’-CGCTGGGCTCTTCCCTGTTCACTCG-3’ |
| d | 5’-CCCGTCCCCCTCCGAGACGCGACC-3’ |
| e | 5’-CGTCTGATCTGAGGTCGCGTCTCGG-3’ |
| f | 5’-CTCTCTCCCGTCGCCTCTCCCC-3’ |

# Supplementary Table 5. Antibodies used in immunoblotting and immunohistochemistry.

| Name | Company |
| --- | --- |
| Anti-β-actin antibody | Beyotime Biotechnology, Shanghai, China |
| Anti-Caspase3 antibody | CST, MA, USA |
| Anti-Cleaved caspase3 antibody | CST, MA, USA |
| Anti-CHK1 antibody | Proteintech, Wuhan, China |
| Anti-CHK2 antibody | Proteintech, Wuhan, China |
| Anti-p-CHK1 antibody | Proteintech, Wuhan, China |
| Anti-p-CHK2 antibody | Proteintech, Wuhan, China |
| Anti-Cyclin D1 antibody | Proteintech, Wuhan, China |
| Anti-puromycin antibody | Sigma-Aldrich, Merck, Darmstadt, Germany |
| Anti-ERK1 antibody | Proteintech, Wuhan, China |
| Anti-ERK2 antibody | Proteintech, Wuhan, China |
| Anti-ERK1/2 antibody | Abcam, Cambridge, UK |
| Anti-p-ERK1/2 antibody | Abcam, Cambridge, UK |
| Anti-ERK5 antibody | Proteintech, Wuhan, China |
| Anti-JNK1 antibody | Proteintech, Wuhan, China |
| Anti-JNK2 antibody | Proteintech, Wuhan, China |
| Anti-JNK3 antibody | Proteintech, Wuhan, China |
| Anti-P38 antibody | Proteintech, Wuhan, China |
| Name | Company |
| Anti-p-P38 antibody | Proteintech, Wuhan, China |
| Anti-RAS antibody | Abcam, Cambridge, UK |
| Anti-B-RAF antibody | Abcam, Cambridge, UK |
| Anti-RAF1 antibody | Abcam, Cambridge, UK |
| Anti-MEK1/2 antibody | Abcam, Cambridge, UK |
| Anti-EFNA1 antibody | Abclonal, Wuhan, China |
| Anti-FBL antibody | Abcam, Cambridge, UK |
| Anti-Ki-67 antibody | Abcam, Cambridge, UK |
