# Supplementary material for: Small nucleolar RNA SNORD13H suppresses tumor progression via FBL-dependent 2′-O-methylation in hepatocellular carcinoma
Source: Front Genet. 2025 Aug 21;16:1620552. doi: 10.3389/fgene.2025.1620552 (PMC12408296; doi:10.3389/fgene.2025.1620552)
Supplement: Supplementary file 2 [file DataSheet1.docx]

Supplementary Figures


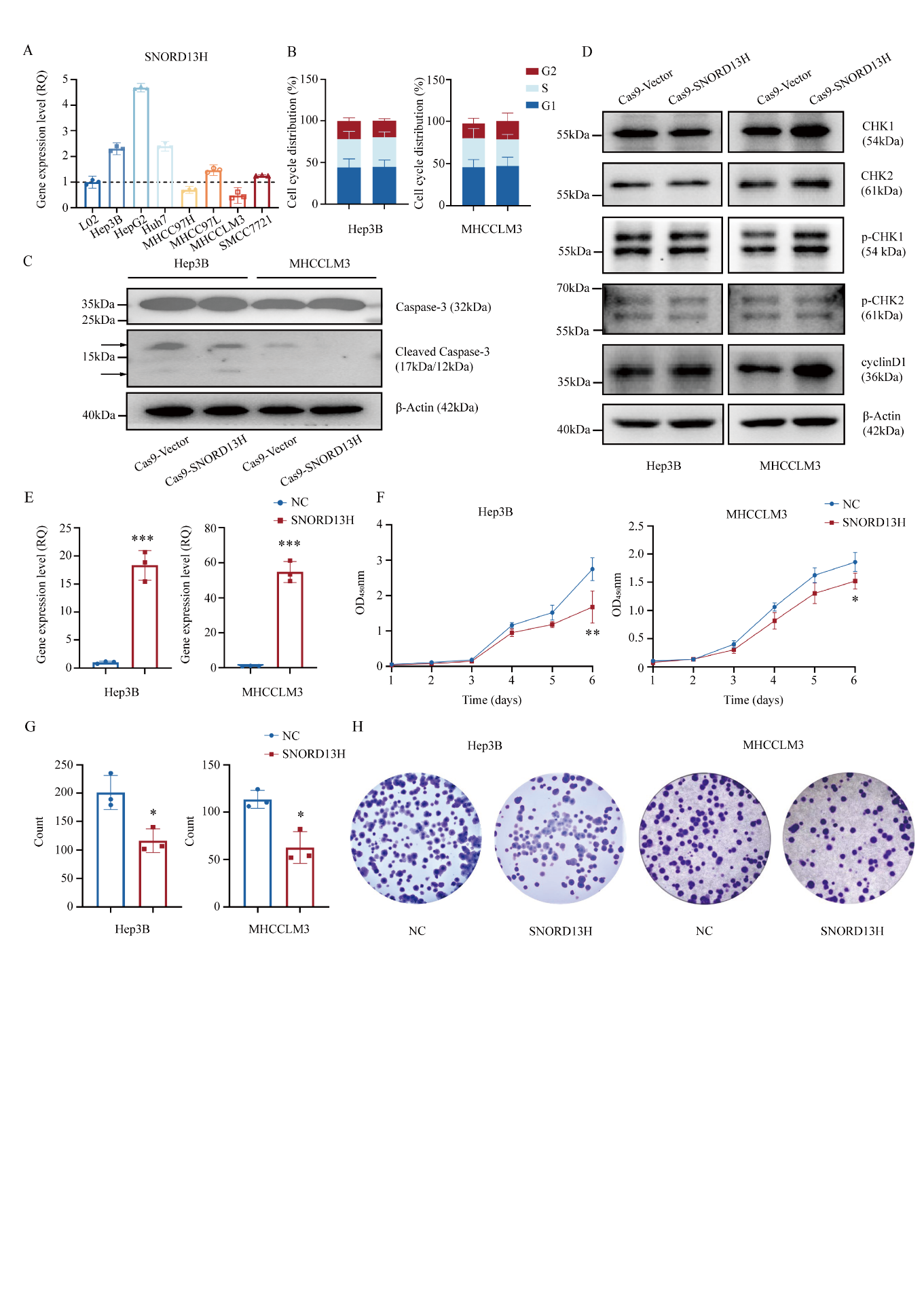


**Supplementary Figure 1.** SNORD13H modulates HCC cell proliferation and apoptosis.

(A) Baseline SNORD13H expression across HCC cell lines (Hep3B, HepG2, etc.) versus immortalized human hepatocyte cell line L-02. Data normalized to L-02 (dashed line=1).

(B) Cell cycle assays of SNORD13H-knockout cells by flow cytometry. Data were processed by Flow Jo software.

(C) Apoptosis suppression. Western blot showed decreased cleaved Caspase3 in SNORD13H-knockout cells (vs. β-Actin).

(D) Cell cycle regulators. Western blot assessed protein alternations of cyclinD1, CHK1, CHK2, p-CHK1 and p-CHK2 in SNORD13H-knockout cells with β-Actin loading controls.

(E) Validation of SNORD13H overexpression by Q-PCR in Hep3B (​​left, p = 0.0004) and MHCCLM3 (​​right, p = 0.0001). Bars: mean±SD (Student’s t-test).

(F) Proliferation suppression. CCK8 assays confirmed reduced growth and proliferation in SNORD13H-overexpressing cells. Results were displayed as absorbances at a wavelength of 450nm. Dots and bars: mean±SD. Statistical significance was analyzed by two-way ANOVA and Student’s t-test. Left, Hep3B (**, p=0.0059); right, MHCCLM3 (*, p=0.0170).

(G-H) Clonogenic inhibition. (G)​​ Quantification of colony formation assays (Hep3B: left, *p = 0.0156; MHCCLM3: right, *p = 0.0104). (H)​​ Representative images showing fewer/smaller colonies in SNORD13H-overexpressing groups. Data were processed by Image J software. Each dot represented one independent experiment and lines indicated mean±SD.


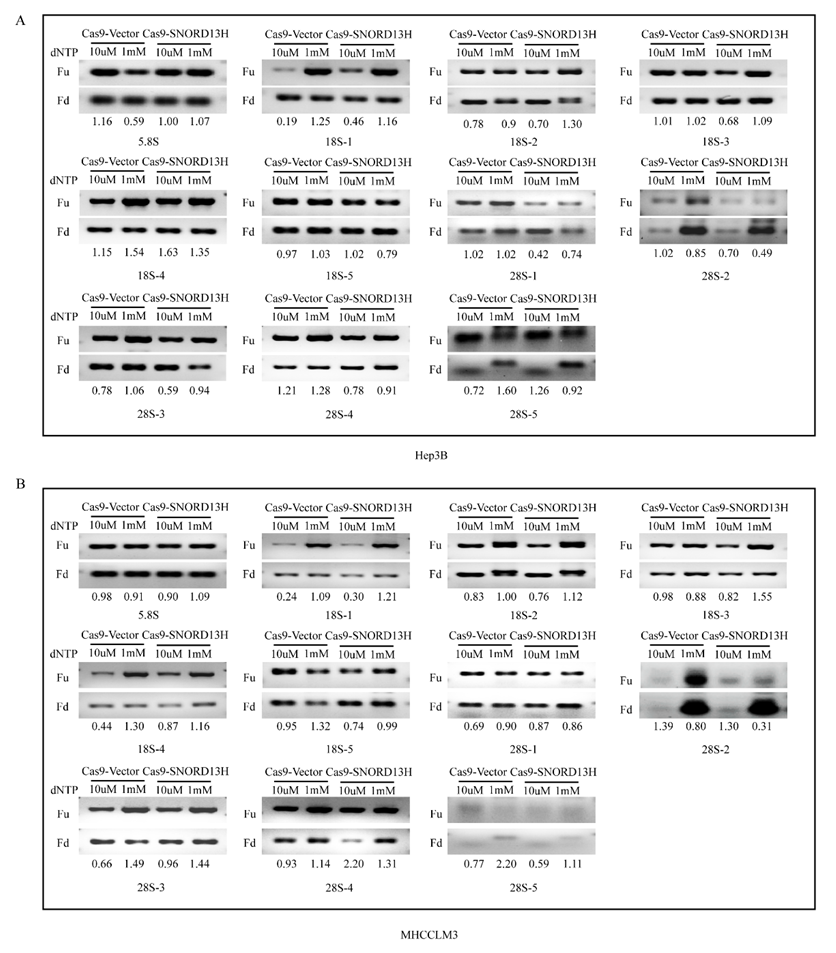


**Supplementary Figure 2**. SNORD13H reduces 2’-O-methylation of rRNAs.

(A-B) RTL-P analysis​​ of rRNA 2’-O-methylation in ​​(A)​​ Hep3B and ​​(B)​​ MHCCLM3 SNORD13H-knockout cells. 2’-O-methylation levels at specific sites in 5.8S, 18S and 28S rRNA were quantified. Control: Normalized to vector-transfected cells; downstream amplicons (Fd) served as loading controls. Results were calculated by Image J software. Statistical results were shown in Figure 3B.


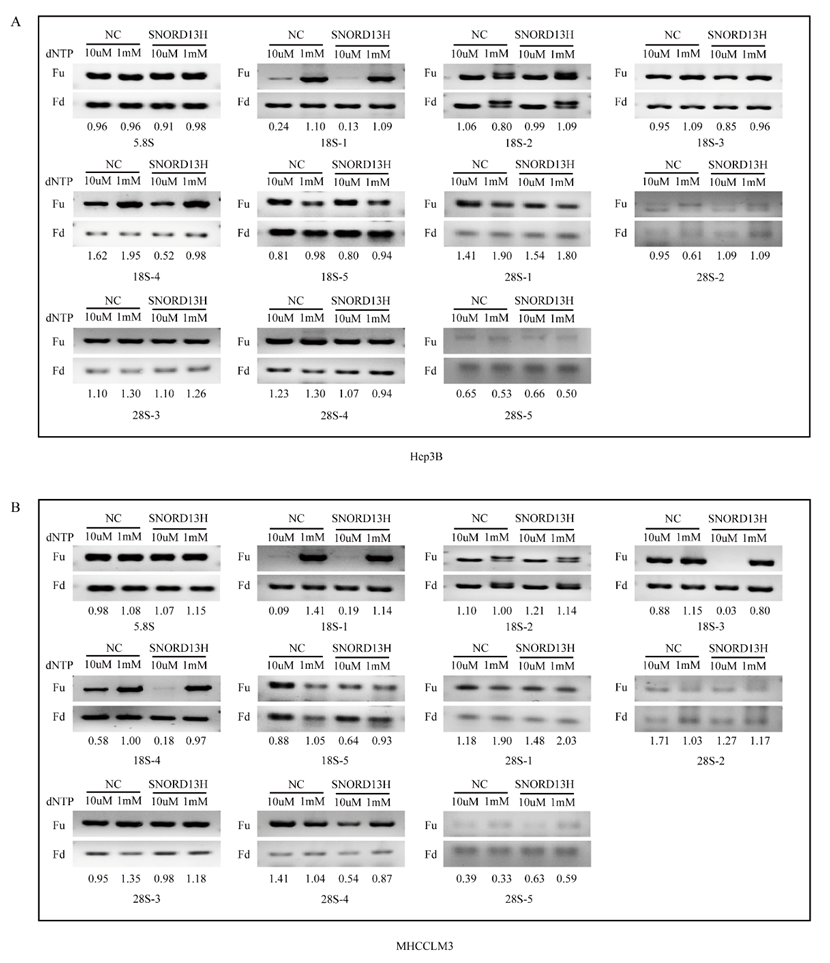


**Supplementary Figure 3.** SNORD13H overexpression enhances rRNA 2’-O-methylation in HCC.

(A-B) RTL-P analysis of rRNA 2’-O-methylation in ​​(A)​​ Hep3B and ​​(B)​​ MHCCLM3 cells overexpressing SNORD13H. 2’-O-methylations were quantified at specific sites in 5.8S, 18S and 28S rRNA. Data was normalized to vector controls (NC); downstream amplicons (Fd) served as loading controls. Data were processed by Image J software. Statistical results were shown on Figure 3B.


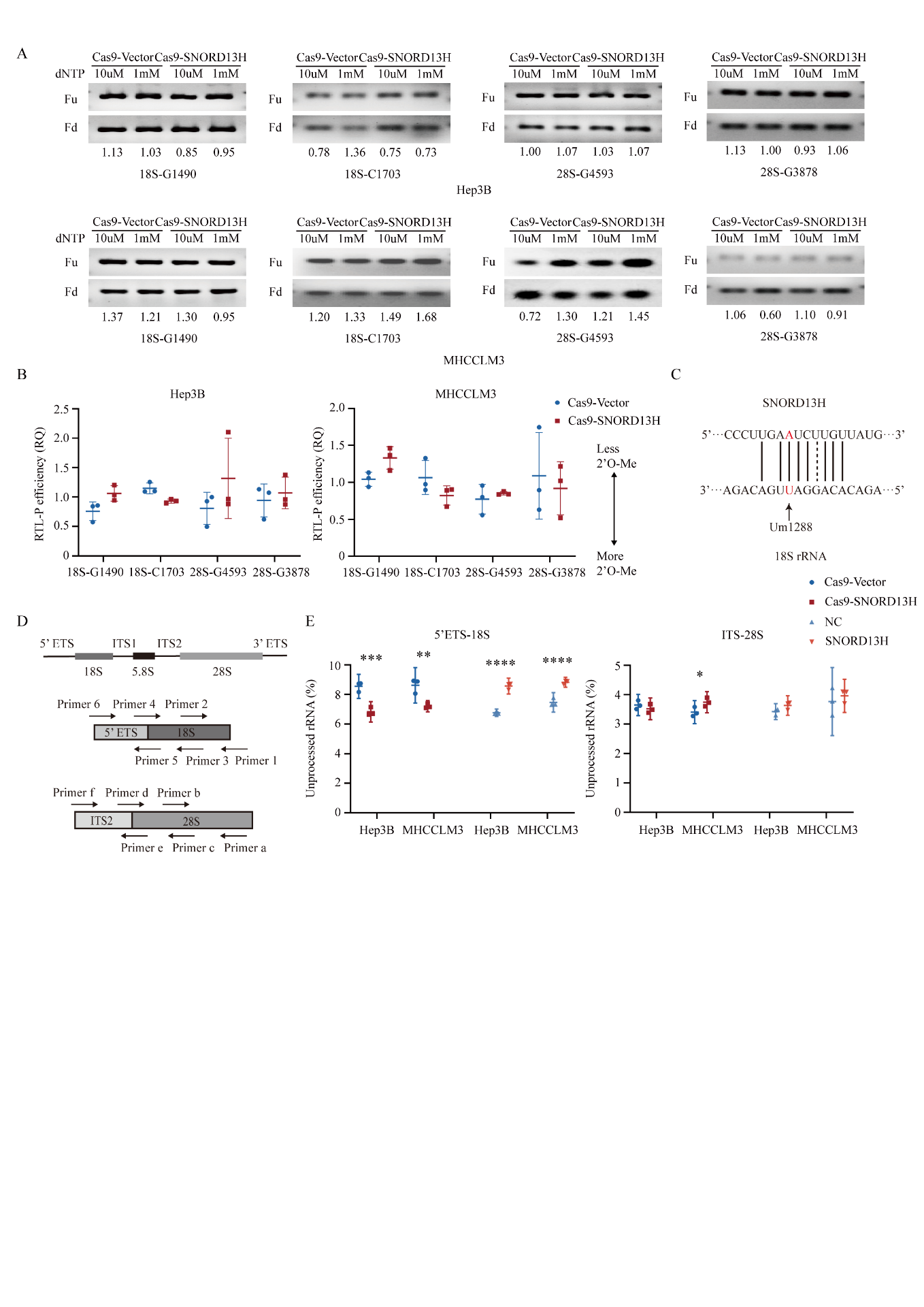


**Supplementary Figure 4.** SNORD13H regulates 18S rRNA maturation through site-specific 2’-O-methylation.

(A-B) RTL-P analysis of rRNA 2’-O-methylation in SNORD13H-knockout cells. ​​(A)​​ Representative gel images showing 2’-O-methylation levels at conserved sites in ​​5.8S, 18S​​ and ​​28S rRNA​​. (B)​​ Quantification normalized to control cells. Downstream amplicons (Fd) served as loading controls. Data were processed by Image J. Bars: mean ± SD (Student’s t-test). (*, p<0.05; **, p<0.01; ***, p<0.001 and ****, p<0.0001.)

(C)​​ ​​Predicted base-pairing model​​ between SNORD13H and 18S rRNA. The methylated adenosine (red) is guided by SNORD13H’s antisense element. Solid lines: Watson-Crick pairs; dotted lines: non-canonical interactions.

(D) Experimental design for rRNA processing efficiency assay. Q-PCR primers target precursor-product junctions of 18S and 28S rRNA.

(E) Processing efficiency of 18S and 28S rRNA in SNOD13H knockout and overexpression cells. Bars: mean ± SD (Student’s t-test). (*, p<0.05; **, p<0.01; ***, p<0.001 and ****, p<0.0001.)

**
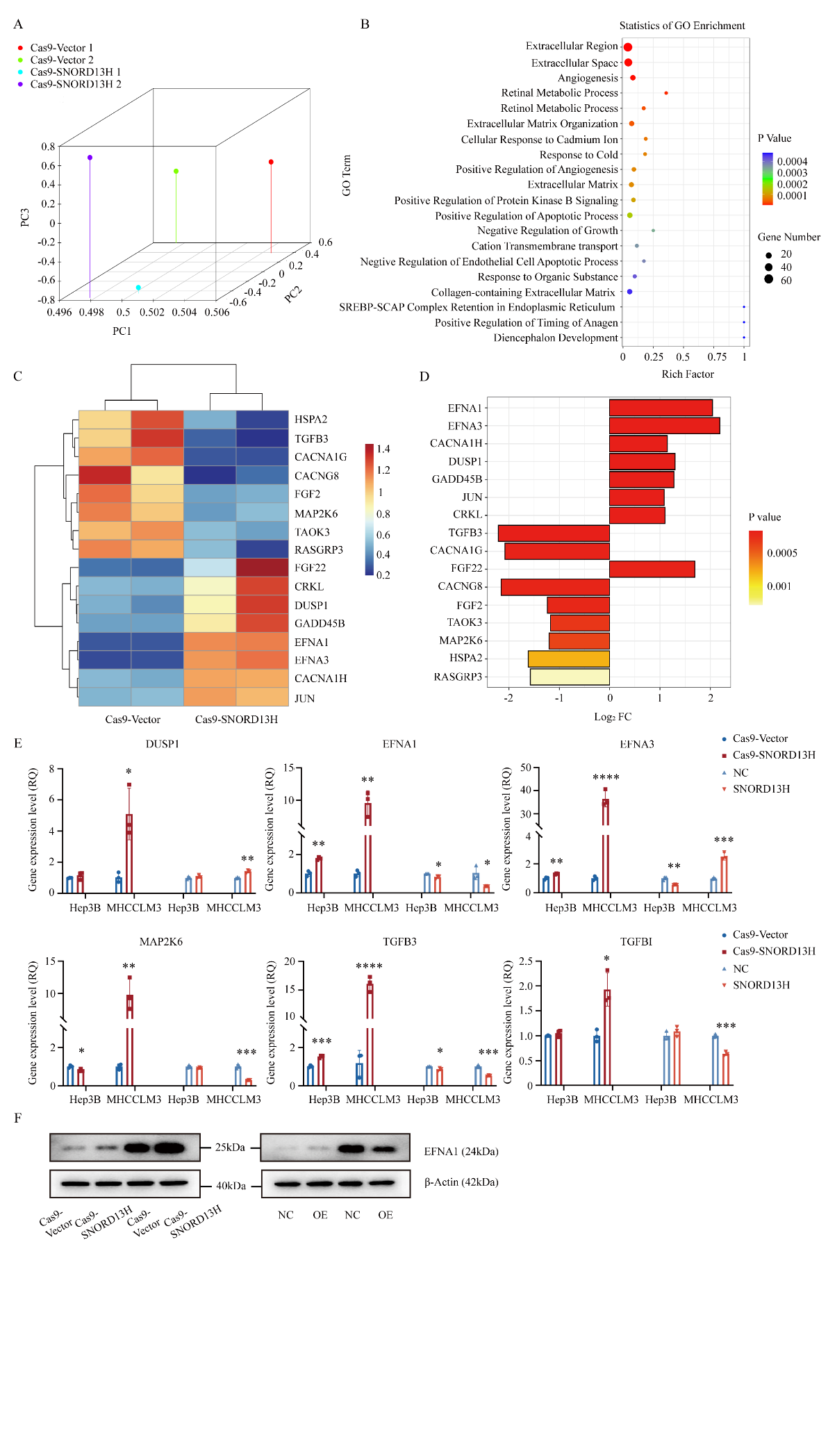
**

**Supplementary Figure 5.** Transcriptomic and functional validation of MAPK pathway activation by SNORD13H deficiency.​

(A)​​ ​​3D principal component analysis (PCA) of RNA-seq profiles​​ showing sample clustering patterns. The plot displays the first three principal components (PC1, PC2 and PC3).

(B)​​ ​​GO enrichment​​ of differentially expressed genes (|log2FC|≥1, FDR<0.05) in RNA-seq. Top 20 significantly enriched terms are shown, colored by p-value and sized by gene count.

(C, D)​​ ​​MAPK-related gene expression. (C) Heatmap of dysregulated MAPK effectors (Log2FC>1.0, p<0.05). Rows: samples; columns: genes. (D) Bar plot of log2FC and p-value for key genes. Bar color: p-value.

(E) Q-PCR validation of MAPK-related genes in RNA-seq. Data normalized to actin. Bars: mean±SD (Student’s t-test). (*, p<0.05; **, p<0.01; ***, p<0.001 and ****, p<0.0001.)

(F) Western blot assay detecting EFNA1 levels in SNORD13H-knockout cells and SNORD13H-overexpressing cells (vs. β-Actin). EFNA1 was one of genes with differential expression from RNA-seq.


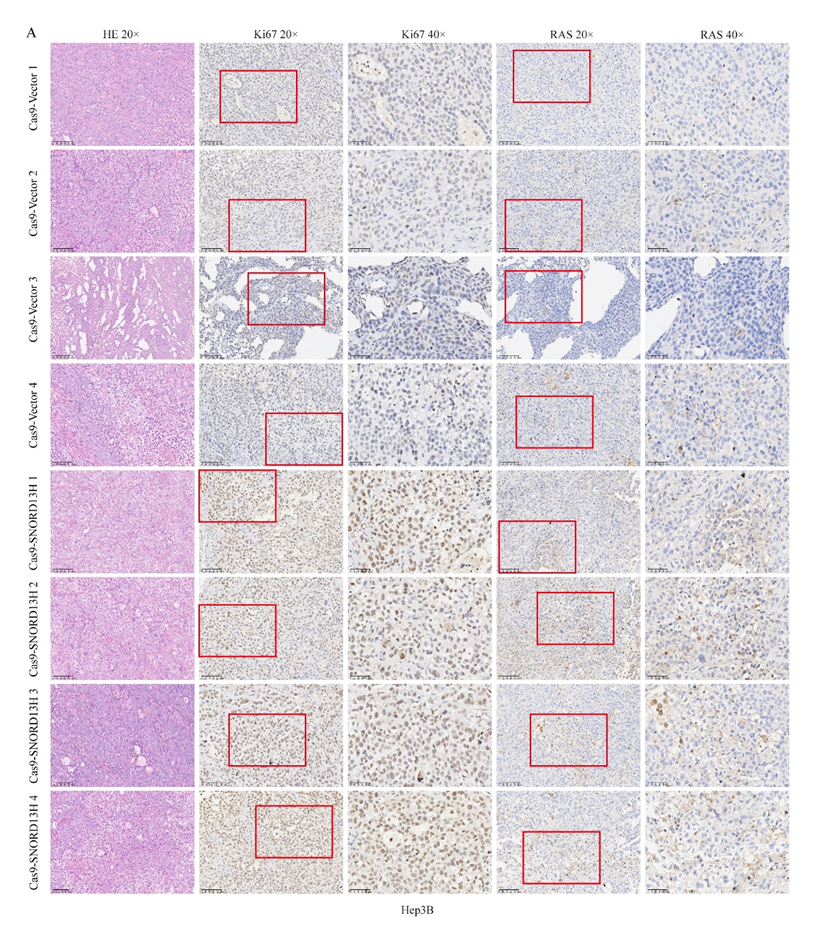


**Supplementary Figure 6.** SNORD13H deficiency enhances Ki67 and RAS levels in Hep3B subcutaneous xenograft tumors.

(A) HE and IHC analysis of Ki67 and RAS in subcutaneous xenograft tumors derived from SNORD13H-knockout Hep3B cells. 40× pictures were enlarged views in red box of corresponding 20× pictures. Scale bars: 100 μm (20×), 50 μm (40×).


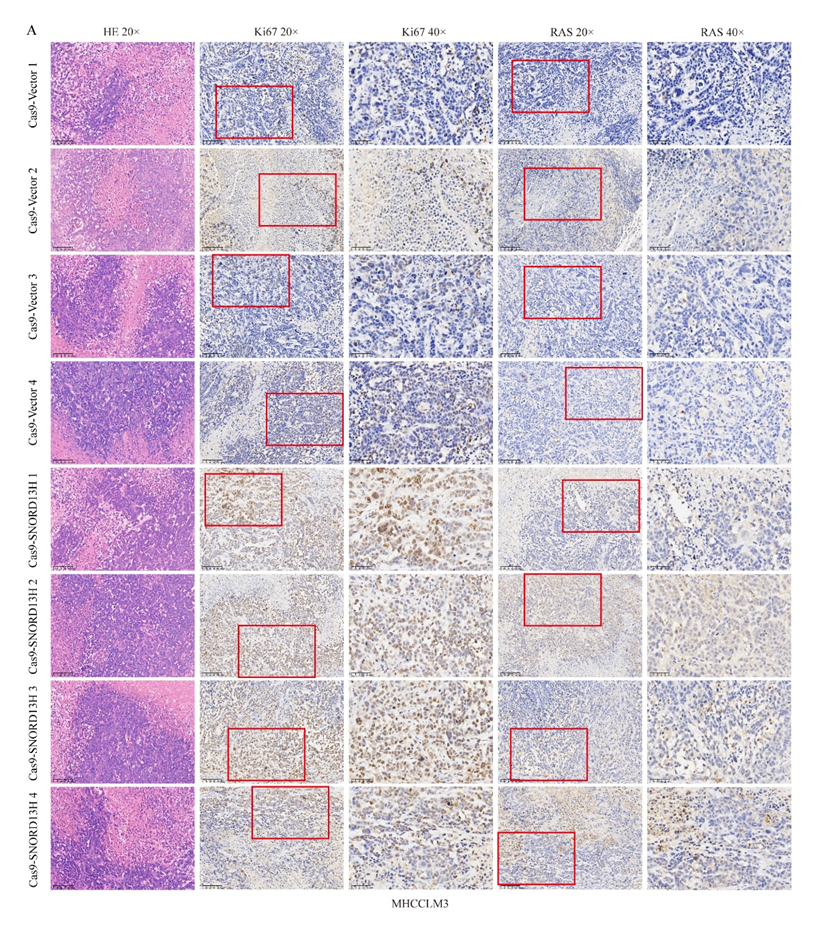


**Supplementary Figure 7.** Decreased SNORD13H increases Ki67 and RAS levels in MHCCLM3 subcutaneous xenograft tumors.

(A) HE and IHC analysis of Ki67 and RAS in subcutaneous xenografts derived from SNORD13H-knockout MHCCLM3 cells. 40× pictures were enlarged views in red box of corresponding 20× pictures. Scale bars: 100 μm (20×), 50 μm (40×).


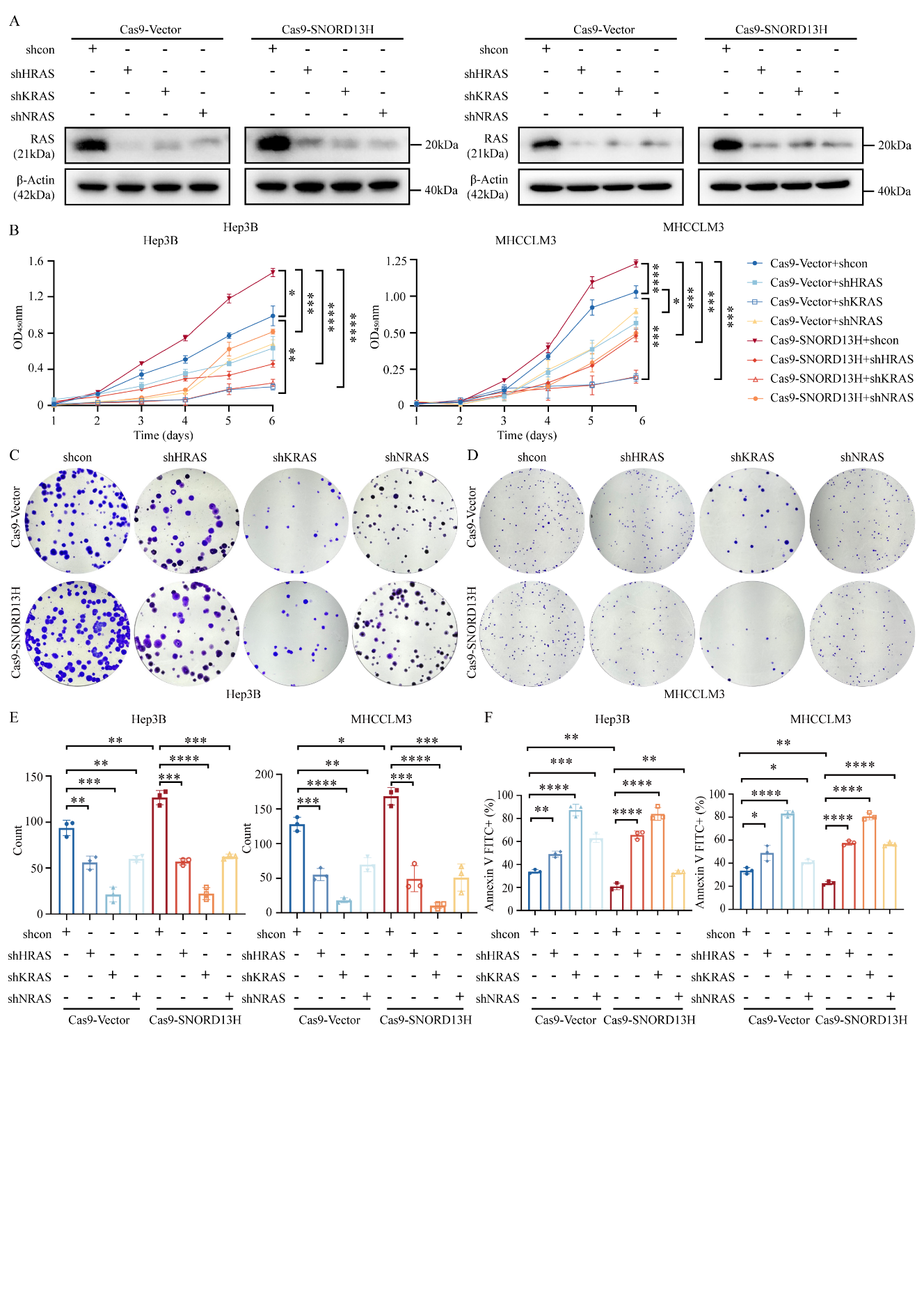


**Supplementary Figure 8.** RAS mediates SNORD13H's tumor-suppressive effects in HCC.​

(A) Western blot validation​​ of RAS knockout in SNORD13H-knockout Hep3B (left) and MHCCLM3 (right) cells. β-Actin served as loading control.

(B) Proliferation rescue​​ by RAS knockout. CCK8 assays showed RAS depletion reversed SNORD13H-knockout-induced hyperproliferation. Statistical significance was calculated using two-way ANOVA and Student’s t-test. Bars: mean±SD.

(C-E) Clonogenic potential​​ analysis. (C-D) Representative colony formation images of SNORD13H/RAS double-knockout Hep3B (C) and MHCCLM3 (D) cells. (E) Quantification demonstrated RAS knockout abolished SNORD13H-knockout-enhanced colony formation. The results were quantified using Image J. Data from 3 independent experiments; bars: mean±SD (t-test). (*, p<0.05; **, p<0.01; ***, p<0.001 and ****, p<0.0001.)

(F) Apoptosis restoration. Flow cytometry (Annexin-V FITC/PI staining) showed RAS knockout rescued apoptosis resistance in SNORD13H-KO cells. Data were processed by FlowJo. Each symbol represented one independent experiment. Bars: mean±SD (Student’s t-test). (*, p<0.05; **, p<0.01; ***, p<0.001 and ****, p<0.0001.)


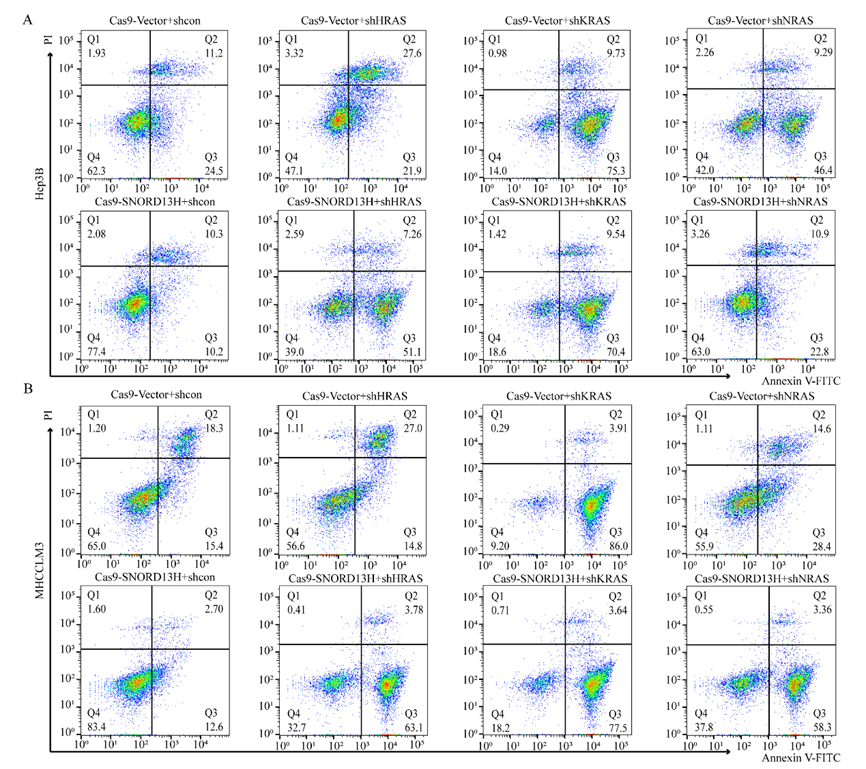


**Supplementary Figure 9.** Interaction between SNORD13H and RAS in regulating HCC cells apoptosis.

(A-B) Flow cytometry analysis of apoptosis in SNORD13H/RAS double-knockout cells. Representative density plots showed Annexin V-FITC/PI staining. Statistical results were displayed in Supplementary Figure 8F. (A), Hep3B; (B), MHCCLM3.


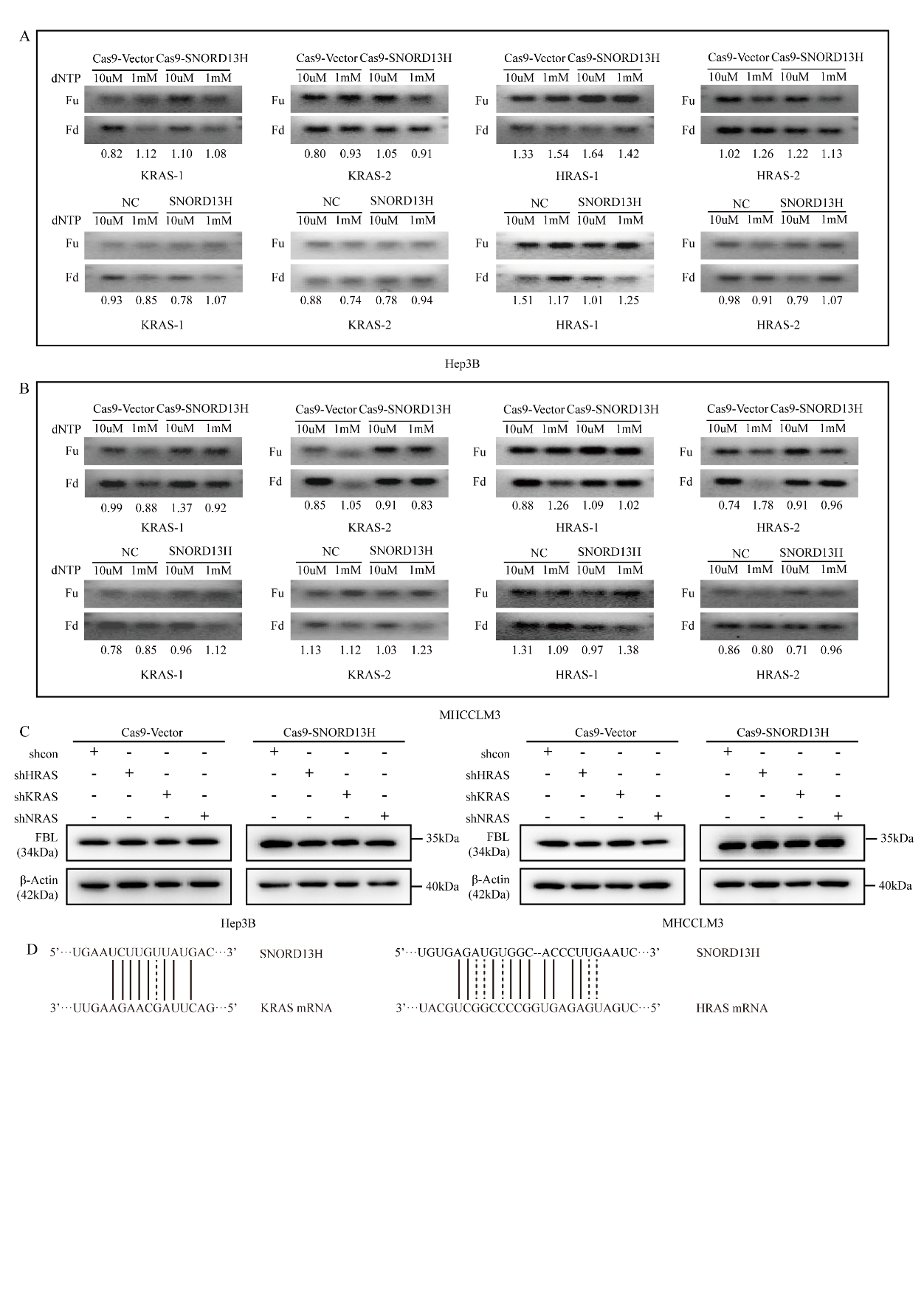


**Supplementary Figure 10.** SNORD13H regulates RAS expression through 2’-O-methylation.

(A-B) RTL-P analysis of 2’-O-methylation in Hep3B (A) and MHCCLM3 (B) cells. Specific segments of HRAS and KRAS mRNA were tested. Statistical results were shown in Figure 5G-H.

(C) Western blot analysis of FBL levels in SNORD13H/RAS double-knockout cells. FBL levels were not affected by RAS loss.

(D) Predicted RNA-RNA interactions. SNORD13H pairs with HRAS and KRAS mRNA. Solid lines: Watson-Crick pairs; dotted lines: non- canonical pairs.
